# Impact and efficacy of systemic antibiotics for peri‐implant diseases treatment: A systematic review and meta‐analysis on clinical and microbiological outcomes

**DOI:** 10.1111/prd.70033

**Published:** 2026-04-01

**Authors:** Gaetano Isola, Alessandro Polizzi, Angela Angjelova, Elena Jovanova, Giuseppe Pizzo, Anton Sculean

**Affiliations:** ^1^ Department of General Surgery and Medical‐Surgical Specialities, Unit of Periodontology University of Catania Catania Italy; ^2^ International Research Center on Periodontal and Systemic Health “PerioHealth” University of Catania Catania Italy; ^3^ Faculty of Dentistry, Ss. Cyril and Methodius University in Skopje Skopje North Macedonia; ^4^ Department of Precision Medicine in Medical, Surgical and Critical Care (me.Pre.C.C.) University of Palermo Palermo Italy; ^5^ Department of Periodontology, School of Dental Medicine University of Bern Bern Switzerland

**Keywords:** amoxicillin, antibiotics, azithromycin, bleeding on probing, clinical trials, meta‐analysis, metronidazole, microbiota, peri‐implant mucositis, peri‐implantitis

## Abstract

**Aim:**

To evaluate the adjunctive effects of systemic antibiotics (SA) on clinical and microbiological outcomes in the treatment of peri‐implant diseases.

**Materials and Methods:**

A systematic review and meta‐analysis were conducted following PRISMA guidelines and registered on PROSPERO (CRD420251059056). Randomized and non‐randomized clinical trials evaluating SA as adjuncts to non‐surgical treatment of peri‐implant mucositis (PM) and to non‐surgical or surgical therapy of peri‐implantitis (PI) were included. Rob2 and MINORS tools were used to assess the risk of bias of included articles.

**Results:**

Eighteen studies were included in the qualitative analysis, of which only nine randomized clinical trials met the criteria for quantitative analysis. For PM treatment, SA did not significantly affect any assessed clinical outcomes (p>0.05). For PI treatment, the meta‐analysis showed that, in both non‐surgical and surgical PI treatment, adjunctive SA resulted in a significant bleeding on probing reduction at 12 months (p=0.007) and a significant probing pocket depth reduction at 12 months (p=0.004). However, no significant improvements in marginal bone level (MBL) were observed. For antimicrobial outcomes, only 2 studies reported significant effects of metronidazole as an adjunct to treatment on reductions in P. gingivalis and T. forsythia up to 12 months.

**Conclusions:**

SA do not provide additional clinical or microbiological benefits in the treatment of PM. In PI, adjunctive systemic antibiotics may offer only limited improvements in selected clinical outcomes and specific peri‐implant pathogens for up to 12 months, without consistent benefits on MBL. However, given the heterogeneity of the available evidence, further high‐quality, long‐term studies are needed.

## 1 INTRODUCTION

Peri‐implant diseases are pathological conditions occurring in tissues around dental implants, plaque‐associated, characterized by inflammation in the peri‐implant mucosa and, if not properly treated, by the subsequent progressive loss of supporting bone.^1^, ^2^, ^3^ Among peri‐implant affections, there is peri‐implant mucositis (PM), when peri‐implant tissues are characterized by bleeding on gentle probing and erythema; swelling and/or suppuration may also be present, or peri‐implantitis (PI) when inflammation of the peri‐implant mucosa and subsequent progressive loss of supporting bone.^4^, ^5^ Despite high implant survival rates, failures may occur over time when disease onset begins with incipient plaque accumulation in PM stage^6^ that evolves, if not properly treated with a targeted peri‐implant treatment, to a frank inflammation of peri‐implant tissues sustained by a pathogenic shift in the subgingival biofilm that determines a subsequent alveolar bone loss and implant loss finally during PI.^7^, ^8^, ^9^

Given the microbial etiopathogenesis of peri‐implant disease conditions, during the last few decades, a plethora of treatments have been developed. Among those, based on the infectious component of diseases, systemic antibiotics (SA) have been widely used in conjunction with peri‐implant mechanical debridement to reduce the microbial load and enhance clinical outcomes.^10^ However, due to the limited availability of standardized quantitative data, their effectiveness remains uncertain.^11^, ^12^ While short‐term improvements in inflammation were observed, the long‐term impact on PI progression appears minimal,^13^ also when adjunct surgery is performed.^14^ Furthermore, concerns regarding antimicrobial resistance, systemic side effects, and the lack of microbial follow‐up persist as challenges to the routine use of antimicrobial agents in PI management.^15^

An increasing number of studies have explored the impact of SA on microbial and clinical outcomes in peri‐implant diseases^16^, ^17^, ^18^, ^19^, ^20^; however, despite growing interest, evidence remains inconclusive regarding their ability to cause significant microbial changes, the clinical relevance of such shifts, and whether their use is justified given global concerns about antimicrobial resistance.^21^, ^22^ However, the absence of standardized outcome measures has contributed to significant heterogeneity in PI clinical trials, limiting the comparability and reliability of findings. To address this, the Implant Dentistry Core Outcome Set and Measurement (ID‐COSM) consensus defined essential outcomes to promote consistency and clinical relevance in future research,^23^ with a central focus on both clinical and microbiological outcomes of peri‐implant tissues.

### 1.1 Peri‐implant microbiota: Composition and dynamics

Although the microbial ecology of periodontal and peri‐implant sites shares some similarities, significant differences exist in composition, particularly under healthy conditions, mainly characterized by a stable and diverse community predominantly composed of Gram‐positive facultative anaerobes such as *Streptococcus* and *Actinomyces species*, which support microbial eubiosis,^24^, ^25^, ^26^, ^27^ determining in several cases high systemic disease risk.^28^

**FIGURE 1:**
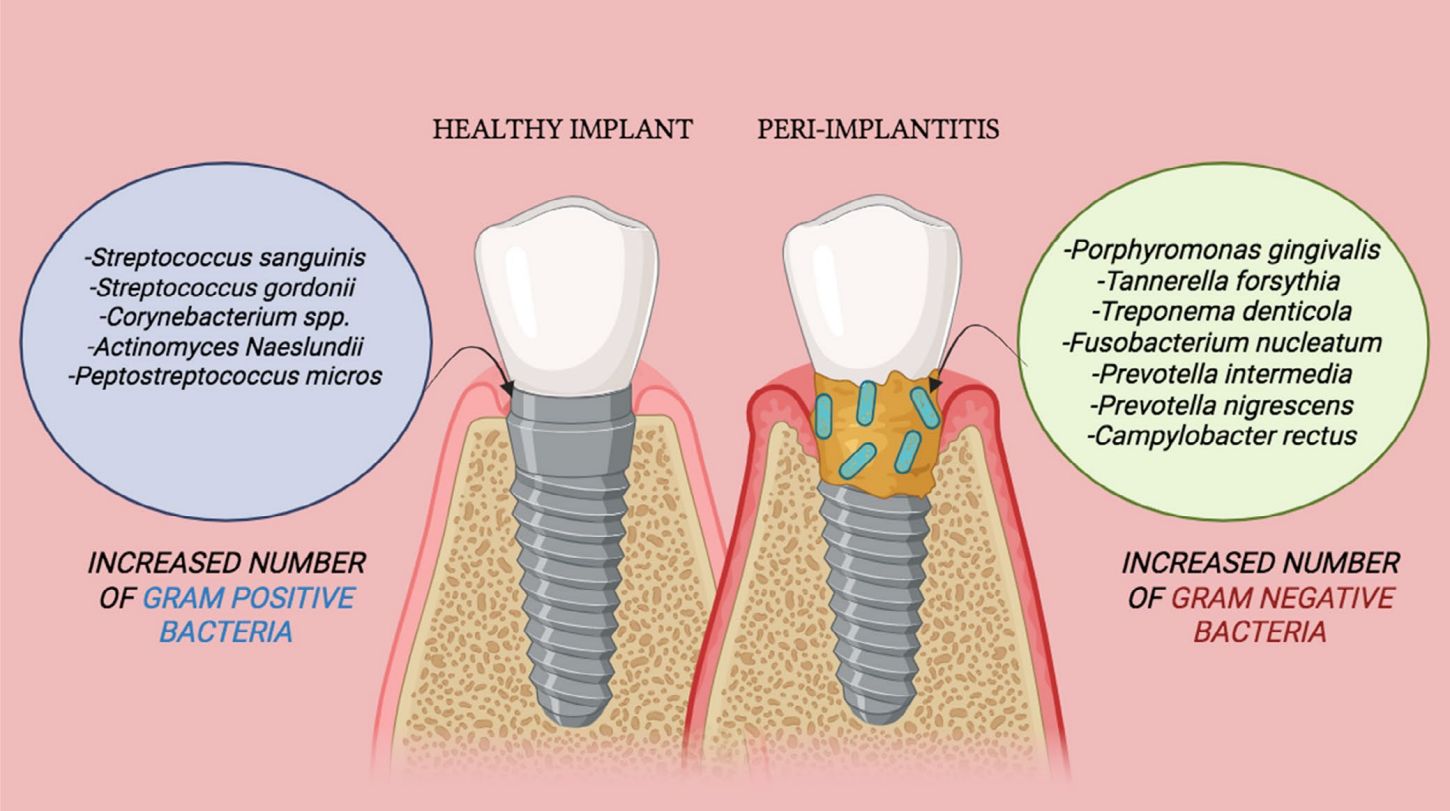
Microbial composition of healthy and diseased peri‐implant sites.

In contrast, healthy peri‐implant sites exhibit significantly lower microbial diversity compared with the subgingival biofilm in natural dentition.^29^, ^30^ Gram‐positive cocci and rods primarily colonize the peri‐implant sulcus, while Gram‐negative anaerobes are present in low abundance.^30^, ^31^ It is well established that small amounts of common periodontal pathogens, such as *Porphyromonas gingivalis* (*P. gingivalis*), *Aggregatibacter actinomycetemcomitans* (*A. actinomycetemcomitans*), *Fusobacterium nucleatum* (*F. nucleatum*), and *Treponema denticola* (*T. denticola*), can be found in peri‐implant sulci even when they appear clinically healthy,^19^, ^32^, ^33^, ^34^ while among the pathogenic organisms implicated in PI are Gram‐negative anaerobes, which play a significant role in tissue inflammation and breakdown.^35^, ^36^ Differences in bacterial populations between healthy peri‐implant sites and PI are summarized in Figure 1.

Similarly, implants and teeth showing signs of PM and gingivitis, respectively, exhibit diverse microbial compositions, suggesting that the direct transmission of the bacterial community from teeth to implants is unlikely. These findings reinforce the idea that although teeth and implants exist in a common environment, their microbial ecosystems remain distinct in both health and disease.^37^, ^38^

### 1.2 Peri‐implant diseases: Definitions and clinical significance

PM is an inflammatory condition affecting the peri‐implant soft tissues, characterized by gentle bleeding on probing (BOP) (>1 spot at a location around the implant) and/or suppuration upon probing (SOP), potentially accompanied by increased probing pocket depth (PPD), yet notably without progressive marginal bone loss (MBL).^1^, ^23^ Importantly, this PM diagnosis excludes any additional bone loss beyond the initial physiological remodeling that may occur during healing after implant placement.^5^, ^39^

If left untreated, PM may progress to PI—a more severe pathological condition characterized by inflammation of the peri‐implant mucosa, accompanied by progressive and irreversible loss of the supporting bone. While a certain degree of crestal bone remodeling (typically 0.5–2 mm) is expected following implant placement and initial loading, any additional radiographic evidence of bone loss beyond this threshold indicates progression of PI, further confirmed by the presence of BOP, PPD, and suppuration.^5^, ^40^, ^41^, ^42^, ^43^

Recent studies estimate that PM affects ~30–47% of patients, while the prevalence of PI ranges from 10% to 20%, depending on whether the data are reported at the implant or patient level.^34^, ^39^, ^44^, ^45^, ^46^

**FIGURE 2:**
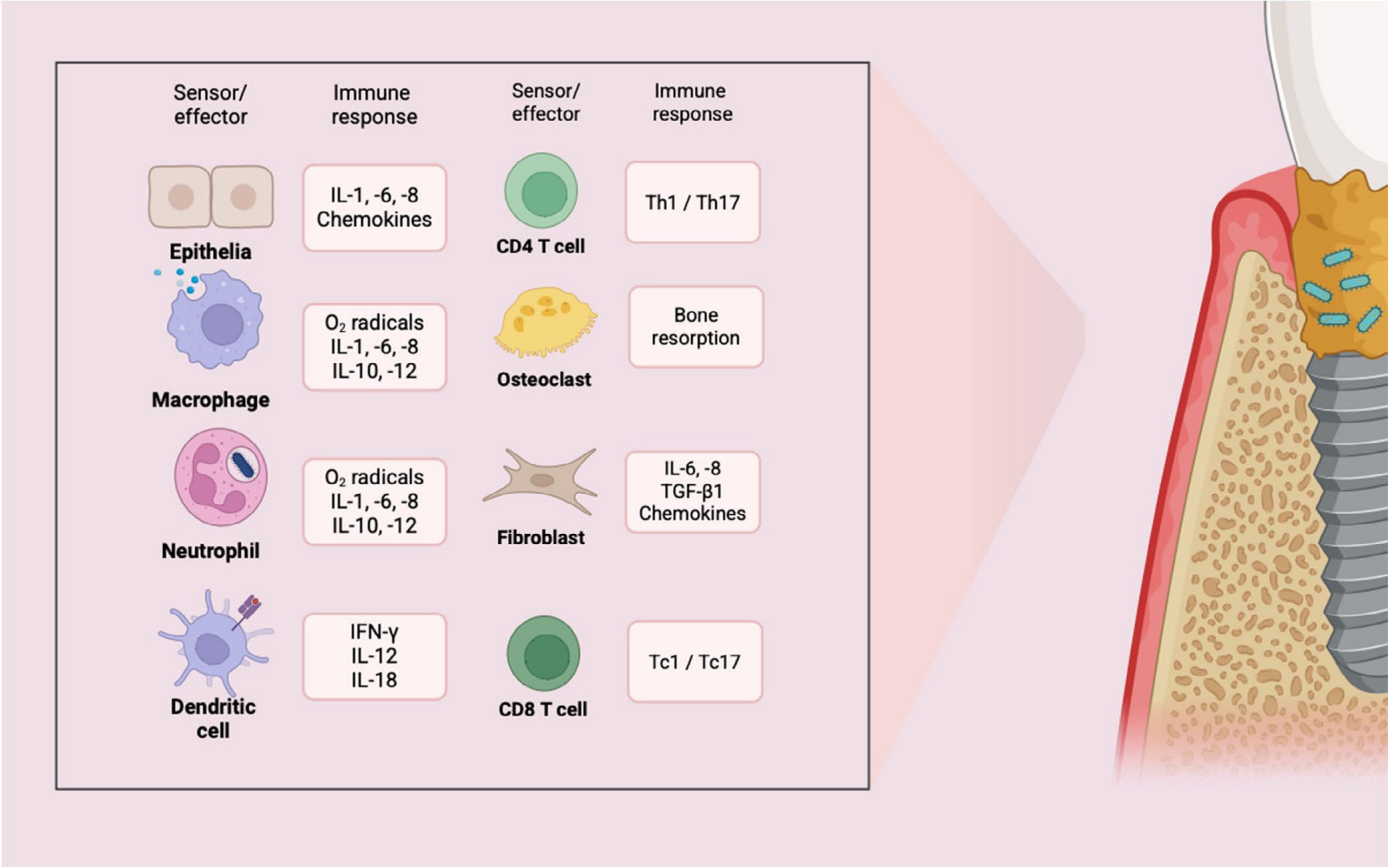
Cellular and molecular immune mechanisms underlying PI.

Peri‐implant sites are more prone to inflammation due to both structural and vascular limitations. Additionally, the soft tissue seal around implants is formed by adaptation rather than true attachment and blood flow is limited to vessels from the outer bone ridge, with minimal vascularization in the connective tissue adjacent to the implant,^47^ favoring the growth of Gram‐negative anaerobic pathogens, such as *P. gingivalis*, *A. actinomycetemcomitans*, and *T. denticola*, which contribute to the onset and progression of PI.^48^, ^49^, ^50^ As the disease progresses to PI, innate immune cells, such as neutrophils, macrophages, and dendritic cells become activated, releasing pro‐inflammatory cytokines like IL‐1 and TNF‐α, which lead to bone resorption and tissue destruction.^51^, ^52^ Figure 2 illustrates the immunopathological mechanisms involved in this progression.

### 1.3 Systemic antibiotics in peri‐implant therapy

**FIGURE 3:**
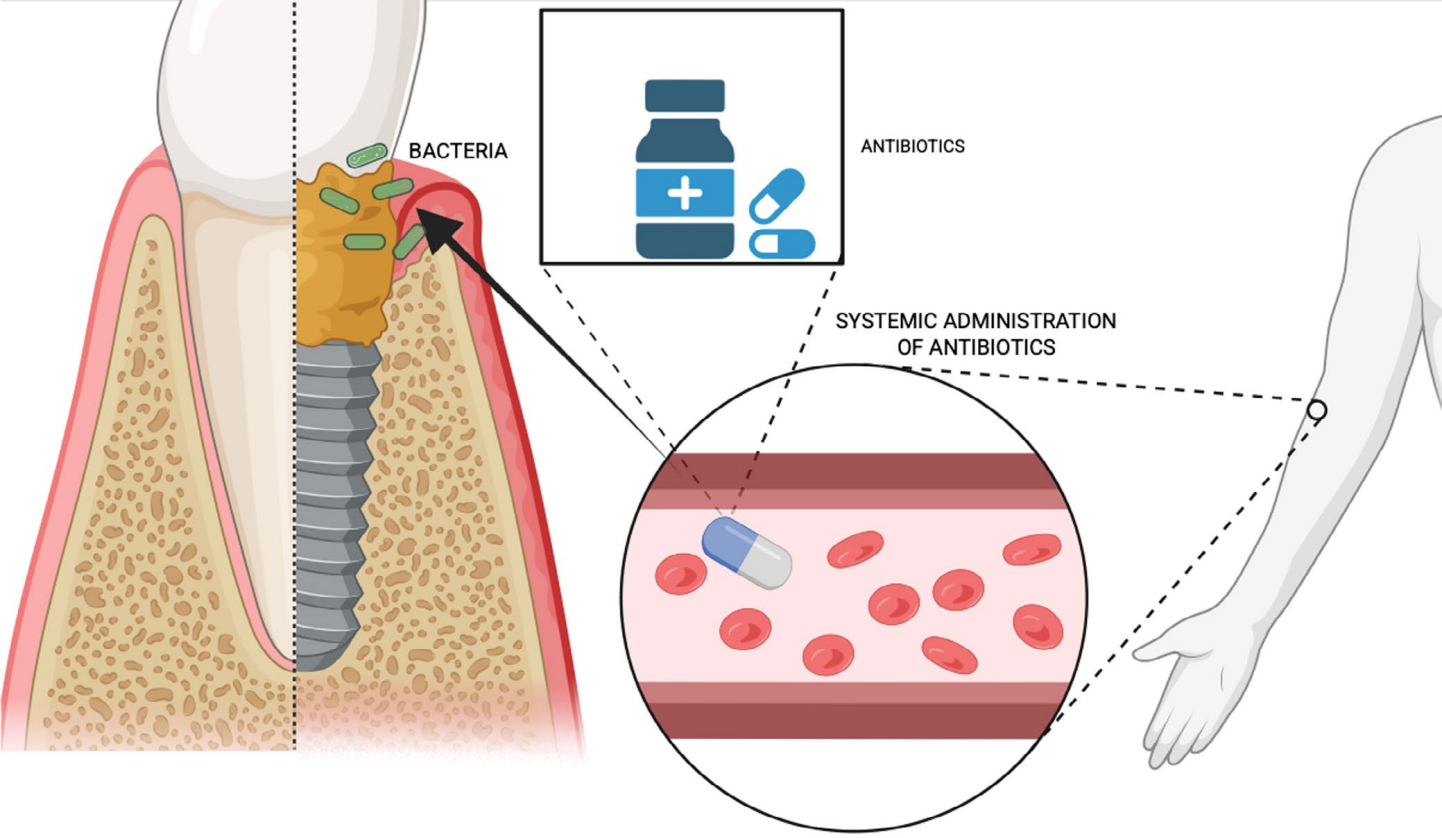
Adjunctive SA therapy targeting peri‐implantitis pathogens.

SA are sometimes included as an adjunct in surgical PI treatment protocols to eliminate identified pathogenic bacteria selectively (Figure 3).^12^ Current PI treatment protocols are largely derived from periodontal therapy evidence and emphasize the resolution of inflammation and the mechanical removal of biofilm from implant surfaces.^53^

The adjunctive administration of antibiotics in PI management can be achieved through systemic administration or local application. While local antibiotic application and chlorhexidine^54^ have demonstrated quite beneficial effects in PM^55^ against specific aerobic and anaerobic bacteria,^54^ in PI treatment without notable adverse effects,^56^ SA provide a less invasive approach with broader antimicrobial coverage and have shown additional clinical improvements when used alongside nonsurgical therapy in several case series and cohort studies.^57^, ^58^, ^59^ Given the pathological characteristics of PI, SA may offer added value when used adjunctively during surgical treatment, particularly in specific patient populations and in implants with particular surface properties.^12^ This benefit is partly due to their extensive penetration and bioavailability in oral tissues, facilitating effective elimination of pathogens from peri‐implant pockets regardless of their specific location.^60^

The systemic administration of antibiotics, such as amoxicillin (AMX), metronidazole (MTZ), and azithromycin (AZT) is commonly employed in peri‐implant therapy due to their complementary antimicrobial spectra, targeting both aerobic and anaerobic bacteria implicated in periodontal and peri‐implant infections, thereby supporting the reduction of microbial load and improving treatment and supportive treatment efficacy.^61^, ^62^ Although both MTZ and AZT, used individually, have been effective in improving key clinical indicators, such as PPD reduction, enhanced CAL, decreased BOP, and pocket resolution, the combination of AMX and MTZ has consistently produced the most favorable therapeutic outcomes.^63^

### 1.4 Clinical outcomes and microbial resistance

SA combined with implant surface debridement has been shown to significantly improve BOP, SOP, PPD, and mucosal recession (MR) in PI patients, particularly at sites with deeper pockets (Table 1).^64^ A recent international consensus report emphasized that the primary objective of PM treatment is to reduce BOP to a clinically acceptable level, specifically ≤1 bleeding site per implant.^23^ In this regard, BOP remains the most reliable clinical indicator for diagnosing PM, with the absence of BOP considered the key indicator of successful resolution following the nonsurgical treatment of PM lesions.^65^, ^66^

**TABLE 1:** Key clinical parameters and clinical relevance in peri‐implant disease based on ID‐COSM consensus.^23^

Parameter	Key insights	Clinical relevance
Bleeding on Probing (BOP)	Most reliable indicator of peri‐implant inflammation. Reduction is a primary treatment objective^23^	Essential for diagnosis and monitoring. Absence indicates resolution of inflammation
Marginal Bone Level (MBL)	Evaluated with standardized intraoral radiographs. Reflects stability or disease progression^23^	Differentiates PM from PI. Key for long‐term assessment
Probing Pocket Depth (PPD)	Reduces with successful treatment, especially with antibiotics in deep sites^56^	Used with BOP and radiographs to assess disease severity
Composite outcome	Concomitant absence of BOP (≤1 spot/implant), SOP, shallow PPD (≤5 mm) and absence of MBL loss	Report the number/proportion of implants/patients, the number/proportion of implants/patients with health/peri‐implant mucositis/peri‐implantitis following the case definition

However, over the past decades, the extensive and indiscriminate use of antibiotics has led to the rapid emergence and widespread dissemination of antibiotic‐resistant bacterial strains, affecting both primary pathogens and opportunistic microbes.^15^, ^67^ Only a minority of trials clearly define primary endpoints and demonstrate a low risk of bias.^68^ While consensus guidelines recommend a 12‐month follow‐up period,^65^ 6‐month short‐term assessments can effectively capture the outcome of the corrective surgical phase and serve as a baseline for maintenance therapy. Nonetheless, long‐term success is influenced by factors like surgical accessibility for biofilm removal and individual risk indicators such as smoking and bone loss.^69^

Despite the growing number of clinical studies, outcomes remain inconsistent due to heterogeneity in study designs, follow‐up durations, and outcome definitions. For the aforementioned reasons, the aim of this study is to comprehensively analyze the most recent research findings to provide updated knowledge and an evaluation of the current evidence on the effects of SA for the treatment of peri‐implant diseases such as PM and PI, with specific attention to their clinical and microbiological outcomes.

## 2 MATERIALS AND METHODS

### 2.1 PICOS question, eligibility criteria, and search strategy

This meta‐analysis has been conducted according to the Preferred Reporting Items for Systematic Reviews and Meta‐analyses (PRISMA) guidelines^70^ and was registered in the PROSPERO database with the registration number CRD420251059056. The following PICOS question has been formulated for this study: “In humans with PM or peri‐implantitis (Population), does adjunctive systemic antibiotic therapy (Intervention), compared with placebo, mechanical debridement/surgery alone or alternative regimens (Comparator), improve clinical peri‐implant outcomes, such as BOP, PPD, SOP, PS (plaque score), MBL, REC (distance from the most apical portion of the crown to the bottom of the periodontal pocket) and/or microbiological outcomes (bacterial load or concentration) (Outcome) as reported by interventional human studies (Study designs)?”

When available from the analyzed studies, peri‐implant clinical parameters were collected and evaluated according to the definitions and recommendations outlined in the ID‐COSM international consensus report by Tonetti et al.^23^ Moreover, randomized clinical trials (RCTs), nonrandomized clinical trials, prospective/retrospective cohort or case‐control studies published in English up to May 31, 2025, evaluating any systemic delivered antibiotic (single or combined) for the management of peri‐implant diseases (PM and PI) and reporting at least one of the predefined clinical or microbiological outcomes were included. Case reports and case series, narrative and systematic reviews, editorials, conference abstracts, and animal or in vitro studies were excluded.

Electronic searching was performed independently by two reviewers on MEDLINE (PubMed), Scopus and Web of Science (WoS) databases. The final search strategy (adapted to database syntax) was (“peri‐implantitis” OR “periimplantitis” OR “peri‐implant diseases” OR “peri‐implant mucositis”) AND (“systemic antibiotic” OR “systemic antibiotics” OR “antibiotic*” OR “amoxicillin” OR “metronidazole” OR “azithromycin” OR “systemic therapy”) AND (“probing pocket depth” OR “PPD” OR “suppuration on probing” OR “SOP” OR “suppuration” OR “bleeding on probing” OR “BOP” OR “plaque score” OR “gingival index” OR “marginal bone loss” OR “marginal bone level” OR “MBL” OR “keratinized tissue” OR “KT” OR “clinical outcomes” OR “microbiol*” OR “bacteria” OR “*Porphyromonas gingivalis*” OR “*Tannerella forsythia*” OR “*Aggregatibacter actinomycetemcomitans*” OR “*Treponema denticola*” OR “*Prevotella intermedia*” OR “*Fusobacterium nucleatum*” OR “total bacterial load”).

### 2.2 Selection of studies and data extraction

Identified records were analyzed by two independent researchers after removing duplicates. The initial screening included a title and abstract assessment. Subsequently, potentially eligible articles were further reviewed in the full‐text versions for adherence to the inclusion criteria. Discrepancies were resolved by discussion or third‐party adjudication. For all eligible papers, two reviewers independently extracted study setting and design, sample characteristics and diagnosis, antibiotic regimen (molecule, dose, duration), comparator, follow‐up, outcomes reported, main findings, and numerical results. Interrater reliability was measured by calculating Cohen's kappa coefficient during screening, which achieved substantial agreement among reviewers (*k* = 0.71).

### 2.3 Risk of bias assessment

Two authors independently evaluated the risk of bias of included articles using the Cochrane Rob2 tool for RCT studies^71^ and the MINORS tool for prospective cohort or retrospective studies.^72^ Risk of bias was assessed using the RoB 2 tool for randomized controlled trials (RCTs) and the MINORS tool for nonrandomized studies. Two reviewers rated each study independently. Disagreements were resolved through consensus with the involvement of a third reviewer if necessary. The RoB 2 assessment included five domains: randomization process, deviations from intended interventions, missing outcome data, measurement of outcomes, and selection of the reported result. The MINORS assessment^72^ included 12 items (1. A clearly stated aim; 2. Inclusion of consecutive patients; 3. Prospective collection of data; 4. Endpoints appropriate to the aim of the study; 5. Unbiased assessment of the study endpoint; 6. Follow‐up period appropriate to the aim of the study; 7. Loss to follow‐up less than 5%; 8. Prospective calculation of the study size; 9. An adequate control group; 10. Contemporary groups; 11. Baseline equivalence of groups; 12. Adequate statistical analyses), scored as 0 (not reported), 1 (reported but inadequate), or 2 (reported and adequate) of the reported results.

### 2.4 Quantitative data synthesis

For quantitative outcomes, where studies provided sufficient numerical data, a meta‐analysis was performed using a random‐effects model. This approach was chosen to account for the expected clinical and methodological heterogeneity among the included studies. Mean differences (MD) and 95% confidence intervals (CI) were calculated for PPD reduction, BOP/SOP reduction, MBL changes (reported as gain or loss), and specific bacterial load reductions (log10 CFU). Heterogeneity across studies was assessed using both the Chi‐squared test and the *I* ^2^ statistic. The *I* ^2^ statistic describes the percentage of variation across studies that is due to heterogeneity rather than chance. Values of 25%, 50%, and 75% were considered to represent low, moderate, and high heterogeneity, respectively. A *p*‐value of <0.05 for the Chi‐squared test was considered indicative of significant heterogeneity. Subgroup analyses were planned where appropriate, primarily to distinguish between PM and peri‐implantitis cases, and to assess the impact of SA regimens at different follow‐up periods. All statistical analyses were conducted using Review Manager (RevMan) Version 5.4.1.

## 3 RESULTS

**FIGURE 4:**
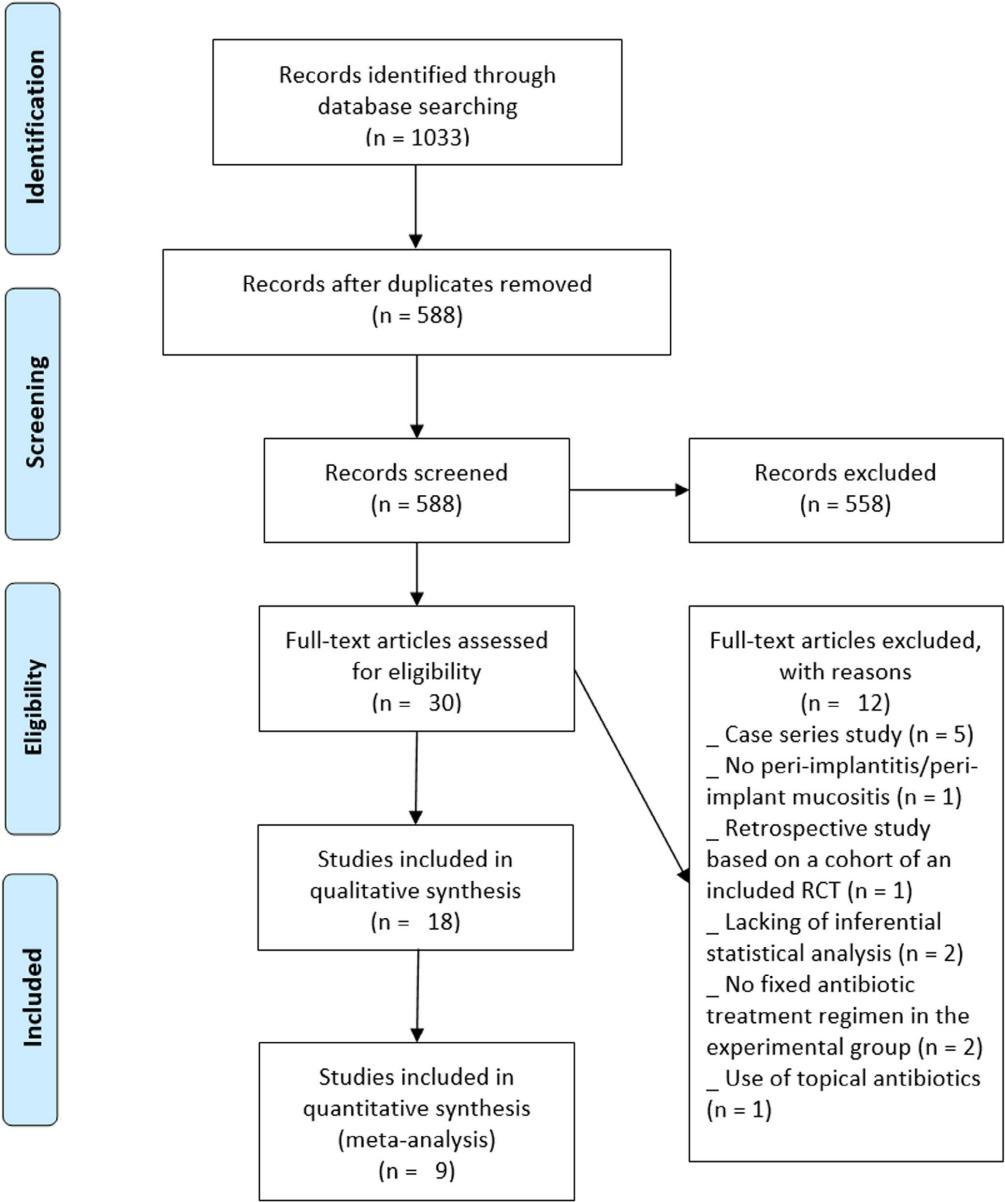
PRISMA flow chart diagram.

The search retrieved 1033 records (PubMed = 273, Scopus = 466, WoS = 294). After title/abstract screening, 30 full‐text articles were assessed, and 18 studies finally fulfilled all eligibility criteria (Figure 1). The reasons for the exclusion criteria for the full text are detailed in Figure 4.

### 3.1 Qualitative synthesis of included articles

Table 2 summarizes core characteristics of the 18 included studies, of which 15 were parallel‐arm RCTs, one prospective cohort, and two retrospective comparative series. Sample sizes ranged from 18 to 100 participants, with diagnoses of PM (*n* = 3) or PI (*n* = 15). Most trials evaluated systemic treatment with AMX + MTZ (*n* = 9) or AZT (*n* = 5) as adjuncts to nonsurgical mechanical (*n* = 12) or surgical debridement (*n* = 6); one study investigated MTZ alone. The last session of recorded follow‐up ranged between 12 weeks and 36 months.

**TABLE 2:** Study and treatment characteristics.

Study	Study design	Sample size, condition	Diagnosis	Intervention	Comparator	Outcomes	Follow‐up	Main results
Al Deeb et al. 2020^73^	Randomized controlled clinical trial	45 smokers	PM	(1) MCD + AZT (500 mg on the first day and 250 mg on the following days 2–4) (2) MCD + PDT	MCD alone	BOP PPD PS *P. aeruginosa* *S. aureus*	6, 12 weeks	Both groups showed significant reductions in clinical and microbiological parameters. Greater BOP reduction in aPDT group
Almohareb et al. 2020^74^	Randomized clinical trial	40	PI	(1) MCD + PDT; (2) MCD + AMX (500 mg TID) + MTZ (400 mg TID) for 7 days	–	PPD, PS, BOP, NPRS, *P. gingivalis*, *T. denticola* and *T. forsythia*	6, 12 months	PDT has shown comparable efficacy to antimicrobial therapy as a supplement to MCD
Alqahtani et al. 2022^75^	Randomized controlled clinical trial	42	PM	(1) MCD + probiotics (*L. reuteri*) (2) MCD + AMX (500 mg TID) for 7 days	MCD alone	PPD, BOP, PS, MBL	3, 6 months	Adjuvant probiotics were more effective than adjunct amoxicillin for the treatment of PM for up to 3 months
Blanco et al. 2022^76^	Randomized controlled clinical trial	32	PI	MCD + MTZ (250 mg TID for 7 days)	MCD + placebo	PPD, CAL, MBL, REC, *P. gingivalis*, *A. actinomycetemcomitans*, *T. forsythia*, *F. nucleatum*, *C. rectus*	3, 6, 12 mo	MTZ induced significant additional improvements in clinical, radiographic, and microbiological parameters after 12 months
Carcuac et al., 2017^77^	Randomized controlled clinical trial	100	PI	Surgical Open Flap + MCD + AMX (2 × 750 mg daily)	Flap surgery + MCD	PPD, BOP/SOP, MBL	12 and 36 months	No reported benefits at 3 years
Carcuac et al., 2016^78^	Randomized controlled clinical trial	100	PI	Surgical Open Flap + AMX (2 × 750 mg daily)	Flap surgery	PPD, BOP, MBL, SOP, total bacterial load, and various bacteria	6, 12 months	AMX improved outcomes only at modified implant surfaces, though overall success rates remained low
De Waal et al. 2021^17^	Randomized controlled clinical trial	57	PI	MCD + CHX + AMX/MTZ (500/500 mg TID for 7 days)	MCD + CHX	PPD, BOP, CAL, MBL, PS, SOP, *A. actinomycetemcomitans*, *P. gingivalis*, *P. intermedia*, *T. forsythia*, *P. micra*, *F. nucleatum* and *T. denticola*	3 months	No significant difference in clinical and microbial outcomes between groups
Gershenfeld et al. 2018^79^	Randomized controlled clinical trial	17	PI	MCD + AZT (500 mg/day for 3 days)	MCD + placebo	BOP, GI, MBL, PS, PPD, SOP, REC, bacterial count	3 days, 1 week, 3 weeks, 3 and 6 months	Patients treated with AZT showed a significant reduction of GI at 6 months
Gomi et al. 2015^80^	Randomized controlled clinical trial	20	PI	MCD + 500 mg/day AZT for 3 days before the procedure	Full‐mouth SRP alone	BOP, PPD, GI, *A. actinomycetemcomitans*, *P. intermedia*, *P. gingivalis*, *T. forsythia* and *T. denticola*	1 week and 1, 3, 6, 9 and 12 months	SA maintained clinical benefits until 9 months follow‐up, but bacterial load increased again at 6 months later
Grundström et al. 2024^16^	Randomized controlled clinical trial	84	PI	(1) Surgical Open flap debridement + CHX + AMX (500 mg TID) + MTZ (400 mg TID) for 7 days; (2) Surgical Open flap debridement + CHX + PMP (800 mg × 2 TID) + MTZ (400 mg TID) for 7 days	Open flap debridement + CHX + placebo	PPD, MBL, BOP, SOP, REC, CAL, *A. actinomycetemcomitans*, *P. gingivalis*, *T. denticola* and *T. forsythia*	12 months	Adjunctive SA showed additional improvements in terms of MBL stability compared with placebo
Hallström et al., 2012^81^	Randomized controlled clinical trial	45	PM	MCD + AZT (500 mg on the first day and 250 mg on the following days 2–4)	MCD alone	BOP, PPD, PS, *F. nucleatum*, *P. intermedia*, *T. forsythia*, *T. denticola*, *P. aeruginosa*, *S. aureus*, and *S. pneumoniae*	1, 3, 6 months	Clinical improvements observed at 6 months
Hallström et al., 2017^82^	Randomized controlled clinical trial	31	PI	Surgical Open flap debridement + CHX + + AZT (500 mg on the first day and 250 mg on the following days 2–4)	Open flap debridement + CHX	PPD, BOP, PS, SOP, and total bacterial load	6,12 months	Open flap debridement had similar benefits to AZT at 1‐year follow‐up
Heitz‐Mayfield et al., 2012^83^	Prospective cohort study	24	PI	Surgical Open flap debridement + CHX + + AMX (500 mg TID) + MTZ (400 mg TID) for 7 days	–	PPD, BOP, PS, SOP, MBL, REC	3, 6, 12 months	The combined protocol was effective up to 12 months follow‐up
Irshad et al. 2021^84^	Retrospective study	46	PI	MCD + CHX + AMX (500 mg TID) + MTZ (400 mg TID) for 5 days	MCD + CHX	PPD, CAL, BOP, SOP, PS, REC, *A. actinomycetemcomitans*, *P. gingivalis*, *P. intermedia*, *T. forsythia*, *P. micros*, *F. nucleatum*, *C. rectus*, and total bacterial load	3 months	Adjunctive SA induced greater REC and improved BOP
Polymeri et al., 2022^53^	Randomized controlled clinical trial	37	PI	MCD + CHX + AMX (375 mg TID) + MTZ (250 mg TID) for 7 days	MCD + CHX	PPD, BOP, SOP, PS	12 weeks	Adjunctive AMX + MTZ did not induced significant benefits compared with MD alone in the management of PI
Ramanauskaite et al., 2024^85^	Retrospective study	49	PI	(1) Surgical reconstructive treatment + AMX (2 g preoperative); (2) Surgical reconstructive treatment + AMX (500 mg TID postoperative for 3 days)	Surgical reconstructive treatment alone	PPD, BOP, PI, SOP, max PPD	6, 12 months	Preoperative and postoperative adjunct antibiotics did not induce additional clinical benefits compared with surgical treatment of PI
Shibli et al., 2019^86^	Randomized controlled clinical trial	40	PI	MCD + AMX (500 mg TID) + MTZ (400 mg TID) for 14 days	MCD + placebo	PPD, BOP, PS, SOP, GI, microbial profiling according to Socransky's microbial complexes	14 days, 3, 6 months, 1 year	Adjunctive SA did not improve clinical and microbiological outcomes compared with control
Tada et al., 2018^87^	Double‐blind randomized controlled clinical trial	30	PI	MCD + AZT (500 mg) for 3 days + probiotics (*L. reuteri*)	MCD + AZT (500 mg) for 3 days + placebo	PPD, BOP, *F. nucleatum*, *P. gingivalis*, *P. intermedia*, *A actinomycetemcomitans*, *T. denticola* and *T. forsythia*	1, 3, 6 months	Probiotics induced inflammation reduction rather than improving peri‐implant bacterial flora

Abbreviations: AMX, Amoxicillin; aPDT, antimicrobial photodynamic therapy; BOP, bleeding on probing; CAL, clinical attachment level; CHX, chlorhexidine; GI, gingival index; MBL, marginal bone level; MCD, nonsurgical mechanical debridement; MTZ, metronidazole; NPRS, numeric pain rating scale; PI, plaque index; PMP, phenoxymethylpenicillin; PPD, probing depth; PS, plaque score; REC, recession; SOP, suppuration on probing; SRP, scaling and root planing; TID, Ter in die. *A. actinomycetemcomitans*, *Aggregatibacter actinomycetemcomitans*, *P. gingivalis*, *Porphyromonas gingivalis*, *P. intermedia*, *Prevotella intermedia*; *T. forsythia*, *Treponema forsythia*; *T. denticola*, *Treponema denticola*; *P. micros*, *Peptostreptococcus micros*; *F. nucleatum*, *Fusobacterium nucleatum*; *C. rectus*, *Campylobacter rectus*; *P. aeruginosa*, *Pseudomonas aeruginosa*; *S. aureus*, *Staphylococcus aureus*; *S. pneumoniae*, *Streptococcus pneumoniae*.

### 3.2 Clinical outcomes

The results on the effects of AS on periodontal clinical outcomes (PPD, BOP, PI, CAL/REC, MBL) are reported in Table 3. Overall, AS determined that mean PPD reductions ranged from 0.8 mm to 3.5 mm across all studies. Of these, attachment loss was evaluated empirically as the mean (relative) CAL^17^ or as the sum of PPD and REC.^76^ However, only two studies reported statistically significant greater PPD reduction when AS were used in comparison to placebo,^76^, ^80^ with a PPD reduction mean value ranging from 0.94 mm^80^ to 2.42 mm.^76^ Similarly, one study reported that SA treatment resulted in a significant SOP reduction at 12‐month follow‐up (MD, 58.7%; *p* < 0.05).^78^ Regarding MBL, although one study^76^ indicated ~ 1 mm of additional bone stability in the SA group, the other six studies did not report any significant MBL stability after treatment compared with controls.

**TABLE 3:** Clinical outcomes from randomized‐controlled clinical trials at baseline and after adjunct systemic antibiotics.

References	Intervention	Antibiotics	Condition	Parameters	Follow‐up	Control	Test	*p*‐value
Blanco et al., 2022^76^	MCD	MTZ	PI	FMBS (BOP, % sites)	Baseline	50.47 (36.92–64.02)^a^	39.85 (24.37–55.32)^a^	NS
					12 months	29.47 (15.82–43.11)^a^	20.77 (13.72–27.82)^a^	NS
Hallström et al., 2012^81^	MCD	AZT	PM		Baseline	24.2 ± 16.7	28.2 ± 20.6	NS
					6 months	18.4 ± 17.4	10.1 ± 6.9	NS
Ramanauskaite et al., 2024^85^	Surgical reconstructive treatment	AMX + MTZ	PI	BOP (% of teeth/implants with BOP >1 site)	Baseline	100	91.7	NS
					12 months	40.0	8.3	NS
					ΔBOP	60.0	83.3	NS
Al Deeb et al. 2020^73^	MCD	AZT	PM	Peri‐implant BOP (% sites)	Baseline	12.3 ± 4.8	15.7 ± 3.9	NS
					12 weeks	8.0 ± 3.7*	10.1 ± 3.1*	NS
De Waal et al., 2021^17^	MCD	AMX + MTZ	PI		Baseline	94.66 ± 9.42	85.96 ± 19.32	NA
					3 months	55.47 ± 31.60	47.37 ± 30.43	NA
					ΔBOP	−39.20 ± 32.31	−38.59 ± 29.60	NA
Gomi et al., 2015^80^	MCD	AZT	PI		Baseline	25.7 ± 2.8	27.9 ± 4.3	0.518
					12 months	19.8 ± 5.7	4.4 ± 0.3	<0.001
Grundström et al., 2024^16^	Open flap debridement + CHX	AMX + MTZ	PI		Baseline	41	45	NS
					Δ(baseline‐12 months)	‐3	−26	
		PMP + MTZ			Baseline	41	38	
					Δ(baseline‐12 months)	−3	−19	
Hallström et al., 2012^81^	MCD	AZT	PM		Baseline	80.0 ± 25.0	82.6 ± 24.4	NS
					6 months	47.5 ± 32.3	27.3 ± 18.8	NS
Shibli et al., 2019^86^	MCD	AMX + MTZ	PI		Baseline	85.0 ± 18.3	86.6 ± 32.2	NS
					12 months	40.3 ± 30.3*	35.6 ± 26.2*	NS
Polymeri et al., 2022^53^	MCD + CHX	AMX + MTZ	PI	Peri‐implant BOP (*n* (%) patients)	Baseline	18 (95%)	17 (94%)	NS
					12 weeks	16 (84%)	14 (78%)	NS
Ramanauskaite et al., 2024^85^	Surgical reconstructive treatment	AMX + MTZ	PI	Peri‐implant BOP (% of implants with BOP >1 site)	Baseline	100	94.7	NS
					12 months	30.8	5.3	NS
					ΔBOP	69.2	89.5	NS
Tada et al., 2018^87^	MCD+ Probiotics	AZT	PI	Peri‐implant BOP (mean number of peri‐implant sites with BOP: 0–6)	Baseline	3.67 ± 1.59	3.20 ± 1.26	NS
					6 months	2.33 ± 1.95	1.53 ± 1.41	NS
Carcuac et al., 2016^78^	Surgical Treatment	AMX	PI	Peri‐implant SOP (% sites)	Baseline	70.3	65.2	NS
					12 months	31.4	6.5	<0.05
De Waal et al., 2021^17^	MCD	AMX + MTZ	PI		Baseline	8.33 ± 16.67	8.33 ± 16.67	NA
					3 months	0.00 ± 0.00	0.00 ± 5.56	NA
					ΔSOP	0.00 ± 16.67	−6.90 ± 12.83	NA
Grundström et al., 2024^16^	Open flap debridement + CHX	AMX + MTZ	PI		Baseline	36	31	NS
					Δ (baseline‐12 months)	−28	−26	
		PMP + MTZ			Baseline	36	38	
					Δ(baseline‐12 months)	−28	−35	
Shibli et al., 2019^86^	MCD	AMX + MTZ	PI		Baseline	5.0 ± 8.0	8.8 ± 8.0	NS
					12 months	5.3 ± 7.1	0*	NS
Ramanauskaite et al., 2024^85^	Surgical reconstructive treatment	AMX + MTZ	PI	Full‐mouth SOP (% of teeth/implants with SOP >1 site)	Baseline	70.0	58.3	NS
					12 months	0.0	8.3	NS
					ΔSOP	70.0	50.0	NS
Polymeri et al., 2022^53^	MCD + CHX	AMX + MTZ	PI	Peri‐implant SOP (*n* (%) patients)	Baseline	12 (63%)	14 (78%)	NS
					12 weeks	3 (16%)*	4 (22%)*	NS
Ramanauskaite A et al., 2024^85^	Surgical reconstructive treatment	AMX + MTZ	PI	Peri‐implant SOP (% of implants with SOP >1 site)	Baseline	61.5	52.6	NS
					12 months	0.0	5.3	NS
					ΔSOP	61.5	47.4	NS
Blanco et al., 2022^76^	MCD	MTZ	PI	CAL (mm)	Baseline	6.08 (5.42–6.74)^a^	7.29 (6.20–8.40)^a^	<0.05
					12 months	5.54 (4.72–6.37)^a^	5.16 (4.48–5.83)^a^ ^,^ *	NS
					ΔCAL	0.53 (−0.33–1.39)^a^	2.14 (0.97–3.30)^a^	<0.05
De Waal YCM et al., 2021^17^	MCD	AMX + MTZ	PI		Baseline	12.45 ± 2.36	12.35 ± 1.68	NA
					3 months	11.49 ± 2.01	11.39 ± 1.62	NA
					ΔCAL	−0.96 ± 1.01	−0.97 ± 0.93	NA
Grundström et al., 2024^16^	Open flap debridement + CHX	AMX + MTZ	PI	CAL at deepest site (mm)	Baseline	8.28 ± 2.58	7.10 ± 1.46	NS
					Δ(baseline‐12 months)	−2.79 ± 2.57	−2.93 ± 2.02	
		PMP + MTZ			Baseline	8.28 ± 2.58	8.0 ± 2.06	
					Δ(baseline‐12 months)	−2.79 ± 2.57	−3.09 ± 2.32	
Shibli et al., 2019^86^	MCD	AMX + MTZ	PI		Baseline	5.9 ± 1.3	7.2 ± 2.6	NS
					12 months	4.4 ± 1.4*	4.2 ± 1.0*	NS
Shibli et al., 2019^86^	MCD	AMX + MTZ	PI	Peri‐implant GI (% sites)	Baseline	10.0 ± 31.6	10.4 ± 41.3	NS
					12 months	4.7 ± 12.5	3.6 ± 12.0	NS
Blanco et al., 2022^76^	MCD	MTZ	PI	MBL (mm)	Baseline	6.00 (5.19–6.81)^a^	6.31 (5.55–7.06)^a^	NS
					12 months	5.05 (4.19–5.92)^a^ ^,^ *	4.16 (3.39–4.94)^a^ ^,^ *	NS
					ΔMBL	0.95 (0.39–1.51)^a^	2.15 (1.51–2.78)^a^	<0.05
Carcuac et al., 2017^77^	Surgical Treatment	AMX	PI		ΔMBL (baseline‐3 years)	0.51 ± 1.87	−0.32 ± 1.35	NA
De Waal et al., 2021^17^	MCD	AMX + MTZ	PI		Baseline	3.03 ± 1.24	2.65 ± 1.61	NA
					3 months	3.08 ± 1.32	2.70 ± 1.65	NA
					ΔMBL	−0.04 ± 0.20	−0.06 ± 0.17	NA
Gershenfeld et al., 2018^79^	MCD	AMX + MTZ	PI		Baseline	6.21 ± 1.57	4.98 ± 1.24	NA
					6 months	6.22 ± 1.39	4.77 ± 1.21	NS
					ΔMBL (%)	−0.80 ± 3.62	3.70 ± 10.45	NS
Grundström et al., 2024^16^	Open Flap Debridement + CHX	AMX + MTZ	PI		Baseline	5.1 ± 2.0	5.1 ± 2.3	NS
					12 months	5.1 ± 2.0	4.2 ± 2.2	
		PMP + MTZ			Baseline	5.1 ± 2.0	5.1 ± 1.8	NS
					12 months	5.1 ± 2.0	4.9 ± 1.8	
Hallström et al., 2017^82^	MCD	AZT	PM		Baseline	4.9 ± 1.7	4.6 ± 1.6	NS
					12 months	4.5 ± 1.5	4.0 ± 1.6	NS
Shibli JA et al., 2019^86^	MCD	AMX + MTZ	PI		ΔMBL (baseline‐12 months)	0.47 ± 0.31	0.41 ± 0.39	NS
Carcuac et al., 2017^77^	Surgical Treatment	AMX	PI	Marginal bone loss >2.0 mm, frequency [*n* (%)]	Δ(baseline‐3 years)	10 (18.9)	3 (4.4)	NA
Gershenfeld et al., 2018^79^	MCD	AMX	PI	PI (0–3)	Baseline	2.4 ± 0.4	2.4 ± 0.3	NA
					Δ(baseline‐3 months)	0.9 ± 0.2	1.8 ± 0.5	NA
Al Deeb et al. 2020^73^	MCD	AZT	PM	Peri‐implant mean PPD (mm)	Baseline	4.5 ± 0.8	4.6 ± 1.1	NS
					12 weeks	4.1 ± 1.0	3.9 ± 1.0*	NS
Blanco et al., 2022^76^	MCD	MTZ	PI		Baseline	6.32 (5.70–6.95)^a^	6.78 (5.95–7.62)^a^	NS
					12 months	5.43 (4.81–6.06)^a^ ^,^ *	4.34 (3.79–4.94)^a^ ^,^ *	<0.05
					ΔPPD	0.89 (0.14–1.64)^a^	2.42 (1.53–3.30)^a^	<0.05
Carcuac et al., 2017^77^	Surgical Treatment	AMX	PI		ΔPPD (baseline‐3 years)	−2.38 ± 2.55	−3.00 ± 2.24	NA
Gomi et al., 2015^80^	MCD	AZT	PI		Baseline	4.35 ± 0.22	4.28 ± 0.85	0.833
					12 months	4.22 ± 0.29	3.34 ± 0.33	0.002
Hallström et al., 2012^81^	MCD	AZT	PM		Baseline	4.6 ± 0.9	4.4 ± 1.0	NS
					6 months	4.1 ± 1.2	3.5 ± 1.1	NS
Hallström et al., 2017^82^	Open Flap Debridement	AZT	PI		Baseline	5.8 ± 0.9	5.7 ± 1.0	NS
					12 months	4.2 ± 1.5	4.0 ± 1.1	NS
Polymeri et al., 2022^53^	MCD + CHX	AMX + MTZ	PI		Baseline	8.00 ± 1.41	7.44 ± 1.38	NS
					12 weeks	6.53 ± 2.59*	5.17 ± 1.92*	NS
					ΔPPD	1.47 ± 1.95	2.28 ± 1.49	NS
Ramanauskaite et al., 2024^85^	Surgical recostructive treatment	AMX + MTZ	PI		Baseline	5.15 ± 1.57	4.28 ± 1.47	NS
					12 months	3.46 ± 1.54	3.15 ± 1.12	NS
					ΔPPD	−1.69 ± 1.56	−1.13 ± 0.99	NS
Shibli et al., 2019^86^	MCD	AMX + MTZ	PI		Baseline	5.5 ± 1.3	7.0 ± 2.6	NS
					12 months	3.8 ± 1.1*	3.9 ± 0.8*	NS
Tada et al., 2018^87^	MCD + Probiotics	AZT	PI		Baseline	4.04 ± 1.14	3.90 ± 0.60	NS
					6 months	3.47 ± 0.95	3.21 ± 0.84	NS
Ramanauskaite et al., 2024^85^	Surgical reconstructive treatment	AMX + MTZ	PI	Full‐mouth mean PPD (mm)	Baseline	5.22 ± 1.67	4.28 ± 1.32	NS
					12 months	3.47 ± 1.58	3.16 ± 1.15	NS
					ΔPPD	−1.74 ± 1.56	−1.13 ± 1.06	NS
Carcuac et al., 2017^77^	Surgical Treatment	AMX	PI	Peri‐implant PPD [frequency >5 mm, *n* (%)]	3 years	20 (37.7)	22 (32.4)	NA
Ramanauskaite et al., 2024^85^	Surgical reconstructive treatment	AMX + MTZ	PI		Baseline	6 (46.2)	5 (26.3)	NA
					12 months	3 (23.1)	2 (10.5)	NA
Ramanauskaite et al., 2024^85^	Surgical reconstructive treatment	AMX + MTZ	PI	Full‐mouth PPD [frequency >5 mm, *n* (%)]	Baseline	5 (50.0)	3 (25.0)	NA
					12 months	2 (20.0)	1 (8.3)	NA
Carcuac et al., 2016^78^	Surgical Treatment	AMX	PI	Peri‐implant PPD (at the deepest site, mm)	ΔPPD (baseline‐1 year)	−1.95 ± 1.81	−3.49 ± 1.54	<0.05
Grundström et al., 2024^16^	Open Flap Debridement + CHX	AMX + MTZ	PI		Baseline	7.55 ± 2.15	7.06 ± 1.19	NS
					Δ(baseline‐12 months)	−3.07 ± 2.23	−3.29 ± 1.47	
		PMP + MTZ			Baseline	7.55 ± 2.15	6.83 ± 1.38	
					Δ(baseline‐12 months)	−3.07 ± 2.23	−3.38 ± 1.72	
Ramanauskaite et al., 2024^85^	Surgical reconstructive treatment	AMX + MTZ	PI		Baseline	7.46 ± 1.33	6.37 ± 1.77	NS
					12 months	5.15 ± 2.41	4.05 ± 1.39	NS
					ΔPPD	−2.31 ± 2.21	−2.32 ± 1.20	NS
Ramanauskaite et al., 2024^85^	Surgical reconstructive treatment	AMX + MTZ	PI	Full‐mouth mean PPD (at the deepest site, mm)	Baseline	6.81 ± 1.55	5.84 ± 1.45	NS
					12 months	4.46 ± 2.58	3.29 ± 1.44	NS
					ΔPPD	−2.34 ± 1.93	−2.55 ± 0.93	NS
Blanco et al., 2022^76^	MCD	MTZ	PI	FMPS (% sites)	Baseline	21.20 (19.41–22.99)^a^	20.92 (18.68–23.16)^a^	NS
					12 months	33.60 (23.89–43.31)^a^	30.77 (23.15–38.39)^a^	NS
Hallström et al., 2012^81^	MCD	AZT	PM		Baseline	36.3 ± 22.8	35.4 ± 25.4	NS
					6 months	32.7 ± 22.1	18.1 ± 10.2	< 0.01
Ramanauskaite A et al., 2024^85^	Surgical recostructive treatment	AMX + MTZ	PI	FMPS (presence/n^a^ probed sites)	Baseline	0.43 ± 0.35	0.39 ± 0.39	NS
					12 months	0.25 ± 0.34	0.25 ± 0.33	NS
					ΔPPD	−0.27 ± 0.37	0.15 ± 0.43	NA
Al Deeb et al. 2020^73^	MCD	AZT	PM	Peri‐implant PS (% sites)	Baseline	45.3 ± 8.1	47.4 ± 10.2	NS
					12 weeks	14.8 ± 5.3*	8.9 ± 2.3*	NS
De Waal et al., 2021^17^	MCD	AMX + MTZ	PI		Baseline	42.11 ± 30.89	42.35 ± 28.02	NA
					3 months	6.88 ± 14.72	8.20 ± 13.28	NA
					ΔPS	−35.23 ± 32.67	−34.15 ± 27.60	NA
Hallström et al., 2012^81^	MCD	AZT	PM		Baseline	22.0 ± 29.2	33.7 ± 35.0	NS
					6 months	17.9 ± 28.7	6.8 ± 13.8	< 0.01
Shibli et al., 2019^86^	MCD	AMX + MTZ	PI		Baseline	61.6 ± 43.7	56.6 ± 43.8	NS
					12 months	61.8 ± 41.6	49.4 ± 30.2	NS
Polymeri et al., 2022^53^	MCD + CHX	AMX + MTZ	PI	Peri‐implant PS (*n* (%) patients)	Baseline	9 (47%)	6 (33%)	NS
					12 weeks	2 (11%)*	3 (17%)	NS
Ramanauskaite et al., 2024^85^	Surgical reconstructive treatment	AMX + MTZ	PI	Peri‐implant PS (presence/n° probed sites)	Baseline	0.37 ± 0.35	0.40 ± 0.41	NS
					12 months	0.19 ± 0.31	0.25 ± 0.34	NS
					ΔPS	−0.23 ± 0.34	−0.01 ± 0.67	NA
Blanco et al., 2022^76^	MCD	MTZ	PI	REC (mm)	Baseline	0.19 (0.02–0.35)^a^	0.44 (−0.06–0.93)^a^	NS
					12 months	0.68 (0.47–0.88)^a^ ^,^ *	0.83 (0.35–1.31)^a^	NS
Grundström et al., 2024^16^	Open Flap Debridement + CHX	AMX + MTZ	PI	REC at deepest site (mm)	Baseline	1.03 ± 1.15	0.53 ± 1.01	NS
					Δ(baseline‐12 months)	0.34 ± 1.2	0.65 ± 1.23	
		PMP + MTZ			Baseline	1.03 ± 1.15	1.51 ± 1.82	
					Δ(baseline‐12 months)	0.34 ± 1.2	0.23 ± 1.83	

*Note*: Values are expressed as mean ± standard deviation (SD) or as mean (95% confidence interval).

Abbreviations: AMX, amoxicillin; AZT, azithromycin; BOP, bleeding on probing; CAL, clinical attachment level; CHX, chlorhexidine; FMBS, full‐mouth bleeding score; FMPS, full‐mouth plaque score; GI, gingival index; MTZ, metronidazole; NA, not available; NPRS, numeric pain rating scale; NS, not significant; PI, plaque index; PMP, phenoxymethylpenicillin; PPD, probing depth; PS, plaque score; REC, recession; SOP, suppuration on probing.

^a^ Values reported as mean (confidence interval – CI).

* Significant *p*‐values compared with baseline.

#### 3.2.1 Peri‐implant mucositis

Studies which analyzed PM finally included for the analysis were Al Deeb et al.,^73^ Alqahtani et al.,^75^ and Hallström et al. 2012.^81^ Al Deeb et al.^73^ using nonsurgical approach plus AZT showed significant reductions in clinical parameters in both test and control groups, with no significant difference in BOP between groups at 3 months. Hallström et al.,^81^ also using AZT as an adjunct to nonsurgical treatment of PM, did not observe short‐term differences and attributed clinical improvements at 6 months to better oral hygiene rather than SA. Alqahtani et al.^75^ in the nonsurgical approach of PM found that AMX was not effective on clinical outcomes at 3 months of the analyzed clinical outcomes.

#### 3.2.2 Peri‐implantitis

For PI, studies such as those Blanco et al.,^76^ Grundström et al.,^16^ and Gomi et al.^80^ included in the analysis reported some positive effects with the adjunctive of SA. Specifically, Blanco et al.^76^ using MTZ in the nonsurgical approach of PI reported statistically significant improvements in CAL and MBL at 12 months, with a mean ΔCAL of 2.14 mm and ΔMBL of 2.15 mm in the test group compared with controls. Gomi et al.^80^ demonstrated maintenance of clinical benefits up to 9 months with AZT plus the nonsurgical approach of PI. However, other studies, such as Carcuac et al.^78^ (flap surgery), De Waal et al.^17^ (nonsurgical approach), Polymeri et al.^53^ (nonsurgical approach), Ramanauskaite et al.^85^ (surgical approach), and Shibli et al.^86^ (nonsurgical approach), often reported no sustained or significant additional benefits of SA on clinical parameters compared with conventional (surgical/nonsurgical) treatment alone for PI.

### 3.3 Microbiological outcomes

The effects of SA on microbiological outcomes around peri‐implant tissues are exposed in Table 4. Regarding microbiological analysis, three studies used quantitative polymerase chain reaction (qPCR)^16^, ^17^, ^76^ and reported extractable data expressed in log10 CFU, consistently demonstrating decreases in certain periodontal pathogens (e.g., *P. gingivalis*, *T. forsythia*, *T. denticola*) after conventional mechanical treatment. However, the majority of comparisons across these studies showed no statistically significant additional benefits from adjunctive SA in reducing these pathogens as an adjunct to nonsurgical/surgical approach. Other studies have also investigated pathogen changes, but they lacked numerical quantitative pooling of reported results.

**TABLE 4:** Microbiological outcomes from randomized‐controlled clinical trials at baseline and after adjunct systemic antibiotics.

References	Condition	Treatment	Antibiotics	Bacteria	Session	Control	Test	*p*‐value
Blanco et al., 2022^76^	PI	MCD	MTZ	*A. actinomycetemcomitans* (log CFU/mL)	Baseline	0 (0, 0)^a^	0 (0, 0)^a^	NS
					12 months	5.4 (−5.5, 5.9)^a^	0 (0, 0)^a^	NS
De Waal et al., 2021^17^	PI	MCD	AMX + MTZ		Baseline	6.08 ± 0.36	7.51	NS
					3 months	6.07 ± 1.85	‐	‐
Grundström et al., 2024^16^	PI	Open flap debridement + CHX	AMX + MTZ		Baseline	3.20 ± 0.51	3.5 ± 0.82	NA
					12 months	3.93 ± 0.82	2.97 ± 0.67	NA
Grundström et al., 2024^16^			PMP + MTZ		Baseline	3.20 ± 0.51	3.39 ± 0.75	NA
					12 months	3.93 ± 0.82	2.26 ± 0.10*	0.012
Blanco et al., 2022^76^	PI	MCD	MTZ	*C. rectus* (log CFU/mL)	Baseline	7.5 (−6.9, 7.9)^a^	7.7 (−7.6, 8.1)^a^	NS
					12 months	6.7 (−5.8, 7.1)^a^ ^,^ *	6.3 (−5.1, 6.7)^a^ ^,^ *	NS
Blanco et al., 2022^76^	PI	MCD	MTZ	*F. nucleatum* (log CFU/mL)	Baseline	7.9 (6.1, 8.3)^a^	7.5 (−6.3, 7.8)^a^	NS
					12 months	7.2 (6.3, 7.5)^a^ ^,^ *	6.4 (−5.2, 6.7)^a^ ^,^ *	NS
De Waal et al., 2021^17^	PI	MCD	AMX + MTZ		Baseline	5.65 ± 0.92	5.78 ± 0.84	NS
					3 months	5.31 ± 1.23	5.11 ± 0.86	NS
De Waal et al., 2021^17^	PI	MCD	AMX + MTZ	*P. micra* (log CFU/mL)	Baseline	5.13 ± 0.94	5.14 ± 0.79	NS
					3 months	4.71 ± 1.15	4.55 ± 0.81	NS
Blanco et al., 2022^76^	PI	MCD	MTZ	*P. gingivalis* (log CFU/mL)	Baseline	8.5 (−8.4, 8.9)^a^	8.1 (6.8, 8.3)^a^	NS
					12 months	7.2 (−6.6, 7.6)^a^ ^,^ *	7.6 (−7.6, 8.1)^a^ ^,^ *	NS
De Waal et al., 2021^17^	PI	MCD	AMX + MTZ		Baseline	6.29 ± 1.09	6.98 ± 1.11	NS
					3 months	5.46 ± 1.88	5.98 ± 2.22	NS
Grundström et al., 2024^16^	PI	Open Flap Debridement + CHX	AMX + MTZ		Baseline	3.22 ± 1.36	4.35 ± 0.93	NA
					12 months	4.25 ± 1.84	2.63 ± 1.24	NA
Grundström et al., 2024^16^	PI		PMP + MTZ		Baseline	3.22 ± 1.36	4.49 ± 1.09	NA
					12 months	4.25 ± 1.84	4.10 ± 0.40	NA
De Waal et al., 2021^17^	PI	MCD	AMX + MTZ	*P. intermedia* (log CFU/mL)	Baseline	5.13 ± 1.26	5.47 ± 1.22	NS
					12 months	5.23 ± 1.61	3.99 ± 2.02	NS
Blanco et al., 2022^76^	PI	MCD	MTZ	*T. forsythia* (log CFU/mL)	Baseline	7.03 (5.8, 7.3)^a^	7.1 (−6.6, 7.5)^a^	NS
					12 months	7.1 (−6.5, 7.4)^a^	5.8 (−5.4, 6.2)^a^ ^,^ *	0.01
De Waal et al., 2021^17^	PI	MCD	AMX + MTZ		Baseline	5.82 ± 0.94	6.22 ± 0.90	NS
					3 months	5.26 ± 1.71	5.32 ± 0.93	NS
Grundström et al., 2024^16^	PI	Open Flap Debridement+ CHX	AMX + MTZ		Baseline	3.54 ± 1.03	3.43 ± 0.97	NA
					12 months	3.72 ± 1.13	3.13 ± 0.86	NA
Grundström et al., 2024^16^	PI	Open Flap Debridement+ CHX	PMP + MTZ		Baseline	3.54 ± 1.03	3.20 ± 1.28	NA
					12 months	3.72 ± 1.13	2.70 ± 0.81*	0.019
De Waal et al., 2021^17^	PI	MCD	AMX + MTZ	*T. denticola* (log CFU/mL)	Baseline	4.96 ± 1.13	5.19 ± 0.75	NS
					3 months	4.39 ± 1.57	4.64 ± 0.80*	NS
Grundström et al., 2024^16^	PI	Open Flap Debridement+ CHX	AMX + MTZ		Baseline	3.79 ± 0.90	3.42 ± 1.14	NA
					12 months	3.80 ± 1.43	2.89 ± 1.26	NA
Grundström et al., 2024^16^	PI		PMP + MTZ		Baseline	3.79 ± 0.90	3.71 ± 1.00	NA
					12 months	3.80 ± 1.43	2.97 ± 0.96	NA

*Note*: Values are expressed as mean ± standard deviation (SD) or as mean (95% confidence interval).

Abbreviations: AMX, amoxicillin; AZT, azithromycin; MTZ, metronidazole; NA, not available.

^a^ Values reported as mean (confidence interval – CI).

* Significant *p*‐values compared with baseline.

#### 3.3.1 Peri‐implant mucositis

Regarding PM, only two studies reported numerical microbiological data results. Of these, Al Deeb et al.^73^ which studied SA as an adjunct to the nonsurgical approach, reported significant reductions in *P. aeruginosa* and *S. aureus* in all groups, with greater reductions in some clinical parameters in the aPDT group. Hallström et al.^81^ evaluated *F. nucleatum naviforme*, *P. intermedia*, *T. forsythia*, *T. denticola*, *P. aeruginosa*, *S. aureus*, and *S. pneumoniae*, but did not find differences between groups when SA were used in adjunct to the nonsurgical approach. The overall data suggest that while mechanical debridement can reduce pathogens in PM, the added SA was not able to significantly reduce peri‐implant pathogens in the short‐ and long‐term.

#### 3.3.2 Peri‐implantitis

For PI, Blanco et al.,^76^ Riben Grundström et al.,^16^ and De Waal et al.^17^ provided quantifiable microbiological data. Blanco et al.^76^ observed that MTZ led to significant additional improvements in microbiological parameters as adjunct to nonsurgical approach, including *P. gingivalis*, *A. actinomycetemcomitans*, and *T. forsythia*, after 12 months. Riben Grundström et al.^16^ showed a statistically significant reduction in *A. actinomycetemcomitans* with Phenoxymethylpenicillin + MTZ at 12 months when SA were used as an adjunct to resective surgery and implant surface decontamination, but not with AMX + MTZ. De Waal et al.,^17^ conversely, reported no significant differences in peri‐implant pathogens between SA and control groups at 3 months for *A. actinomycetemcomitans*, *P. gingivalis*, and *T. forsythia*. Other studies, such as Carcuac et al. 2016^78^ (surgical approach), Gomi et al.^80^ (nonsurgical approach), and Shibli et al.^86^ (nonsurgical approach), also assessed microbiological changes in PI, with mixed results when SA were used as an adjunct to treatments. Gomi et al.^80^ noted an increase in bacterial load after 6 months in the test group, despite initial clinical benefits.

### 3.4 Risk of bias

**FIGURE 5:**
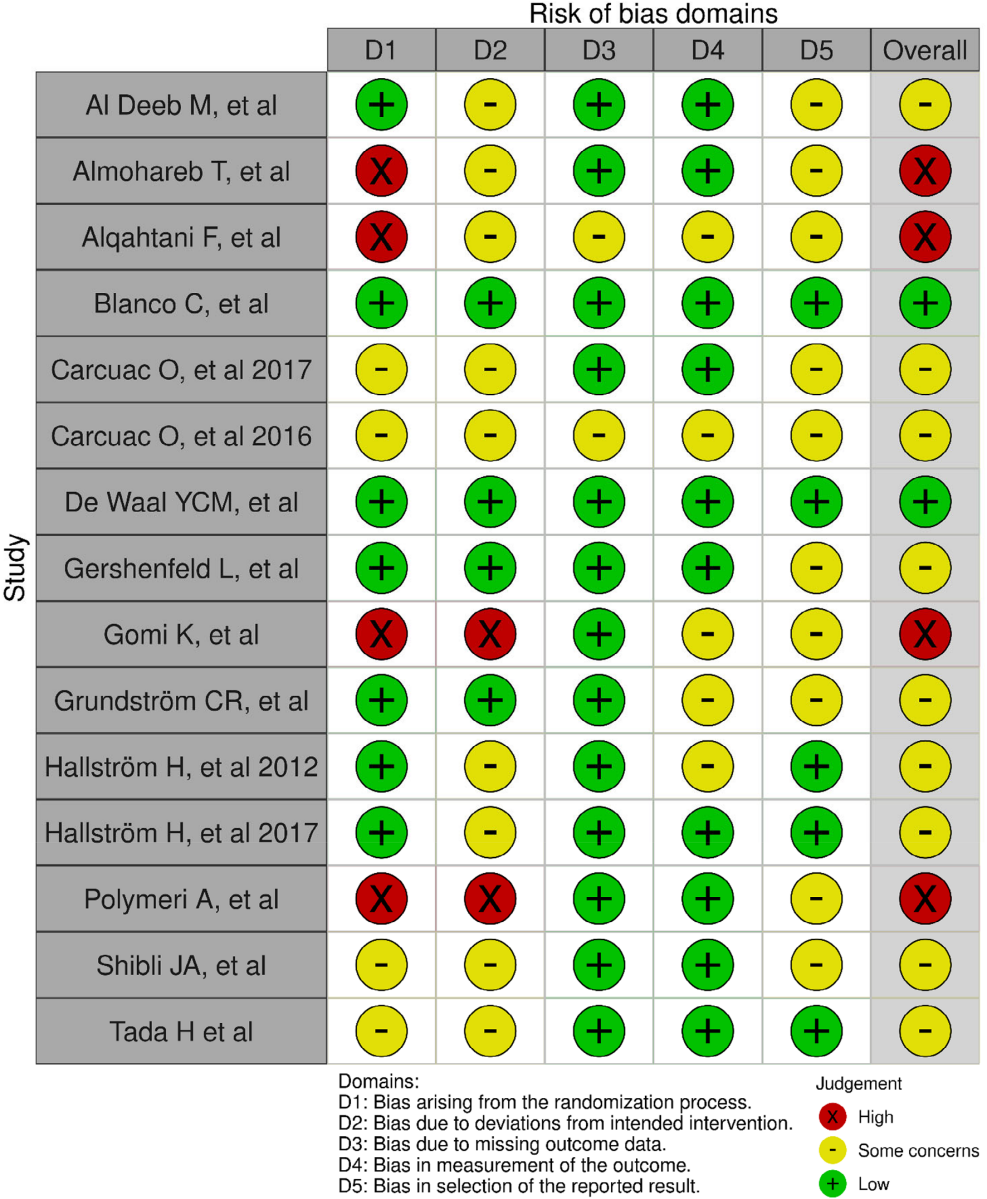
Rob2 risk of bias assessment for randomized clinical trials.

The results of Rob2 analysis are summarized in Figure 5. Among the 15 RCTs, RoB 2 ratings were: low risk (*n* = 2), some concerns (*n* = 3), high risk (*n* = 10). Only two studies were judged to be at low risk of bias.^17^, ^76^ Most other RCTs showed some concerns or high risk in specific domains. In particular, for domain 1 (randomization), some studies did not use or failed to clarify whether allocation concealment was performed,^75^, ^77^, ^78^ or used nonsealed envelopes.^74^ Domain 2 (deviations from intended interventions) was often rated as some concerns, due to the absence of blinding procedures, which may have led to deviations from protocol, particularly in patient adherence or clinical performance. Regarding domain 3 (missing outcome data), most studies were judged at low risk in this domain, as drop‐out rates were low or clearly reported. For the measurement of the outcome (domain 4), some studies were rated as having some concerns because the assessors were probably aware of intervention distribution, and this might influence outcome measures that are based on judgment, potentially biasing the assessment of disease severity and treatment efficacy. Finally, for domain 5 (selection of the reported result), most studies showed some concerns because they did not clarify whether the statistical analysis plan was defined before unblinding the data. Nevertheless, the presence of both significant and nonsignificant (NS) outcomes in the results section suggested a low likelihood of selective reporting.

For the nonrandomized studies, MINORS scores ranged from 12/16 to 20/24 (Table 5). Common limitations included a lack of clearly stated inclusion periods, absence of blinding, and retrospective data collection without explicit standardization. However, some studies demonstrated rigorous reporting of follow‐up and sample size calculations.

**TABLE 5:** MINORS risk of bias assessment for prospective cohort and retrospective studies.

MINORS Item	Heitz‐Mayfield et al.^83^	Irshad et al.^84^	Ramanauskaite et al.^85^
1. A clearly stated aim	1	2	2
2. Inclusion of consecutive patients	1	1	2
3. Prospective collection of data	2	2	2
4. Endpoints appropriate to the aim of the study	2	2	2
5. Unbiased assessment of the study endpoint	0	0	0
6. Follow‐up period appropriate to the aim of the study	2	1	2
7. Loss to follow‐up less than 5%	2	2	2
8. Prospective calculation of the study size	2	2	2
9. An adequate control group	–	2	2
10. Contemporary groups	–	1	1
11. Baseline equivalence of groups	–	0	1
12. Adequate statistical analyses	–	1	2
Total score/MAX score	12/16	16/24	20/24

*Note*: 0, not reported; 1, reported but inadequate; 2, reported and adequate.

### 3.5 Meta‐analysis

**FIGURE 6:**
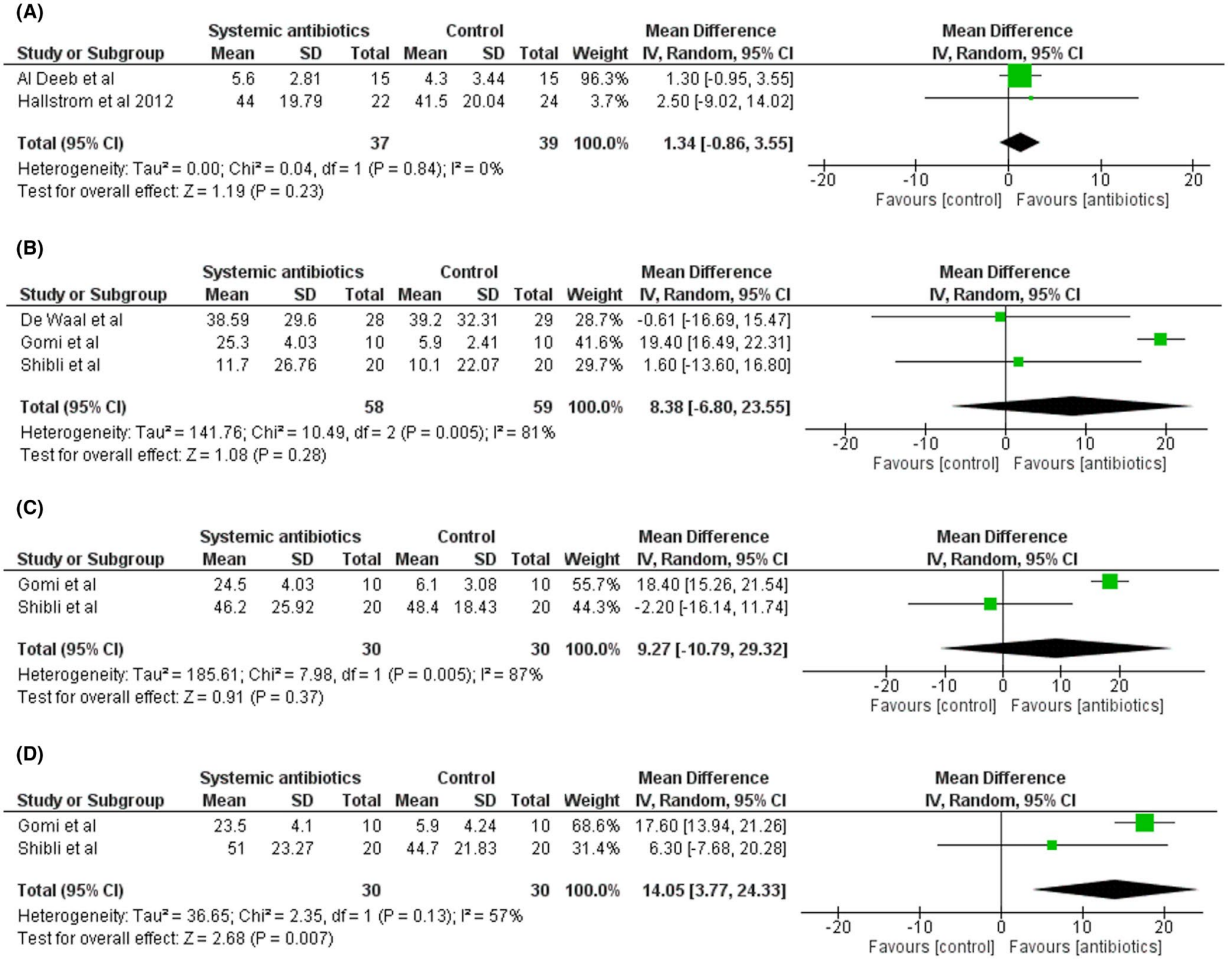
(A) BOP reduction at 3 months in PM; (B) BOP reduction at 3 months in PI; (C) BOP reduction at 6 months in PI; (D) BOP reduction at 12 months in PI.

**FIGURE 7:**
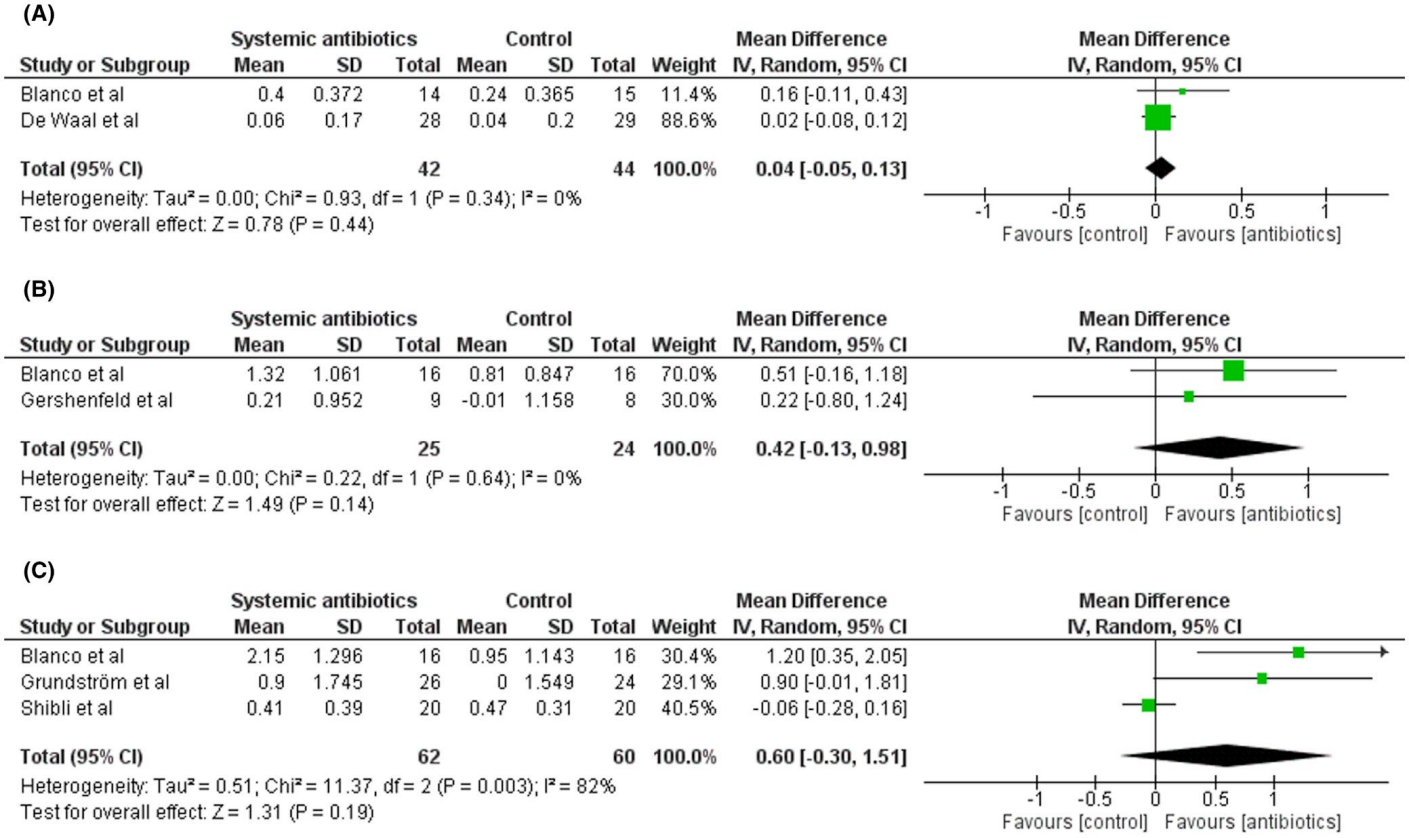
(A) Marginal bone gain at 3 months (peri‐implantitis); (B) Marginal bone gain at 6 months (peri‐implantitis); (C) Marginal bone gain at 12 months (peri‐implantitis).

**FIGURE 8:**
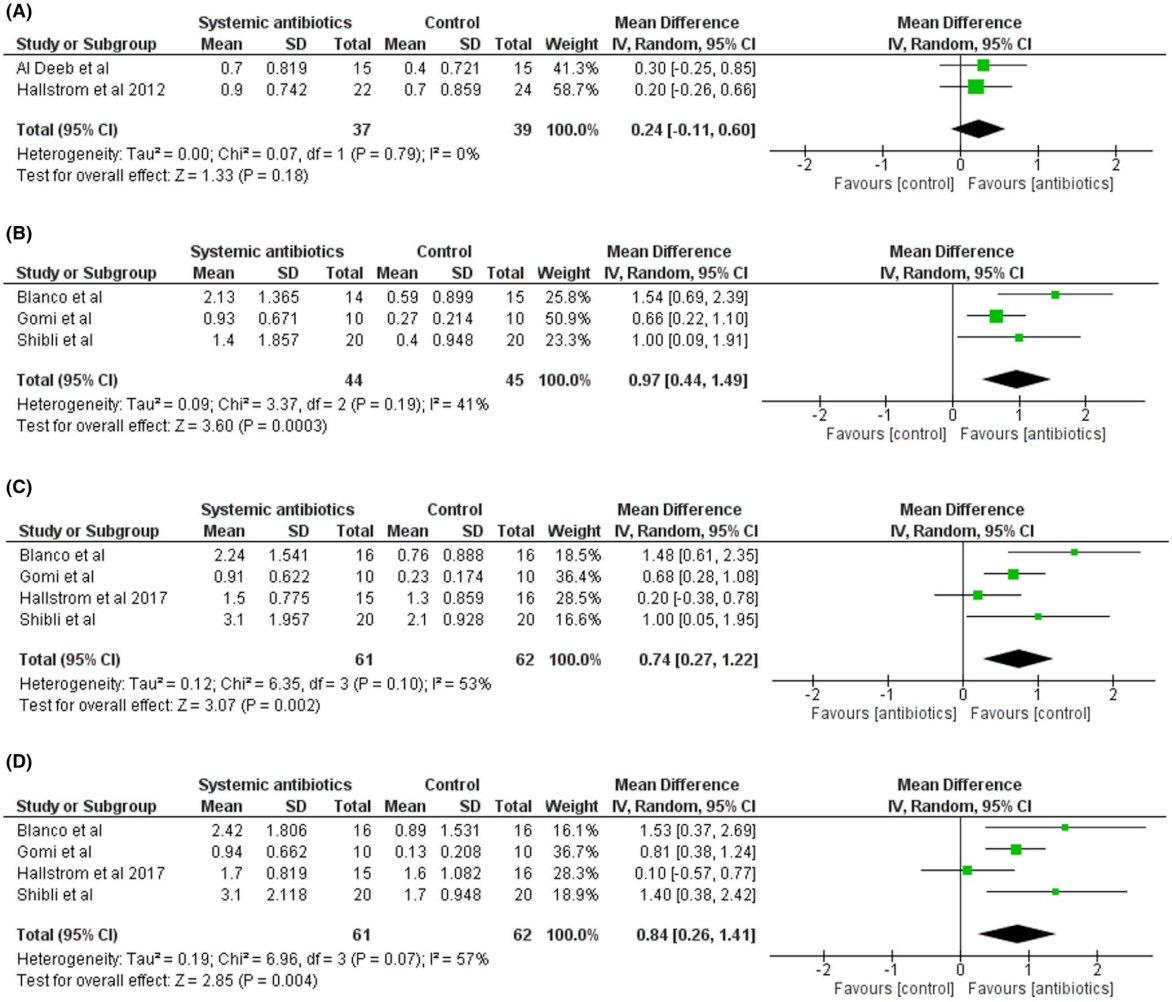
(A) PPD reduction at 3 months in PM; (B) PPD reduction at 3 months in PI; (C) PPD reduction at 6 months in PI; (D) PPD reduction at 12 months in PI.

**FIGURE 9:**
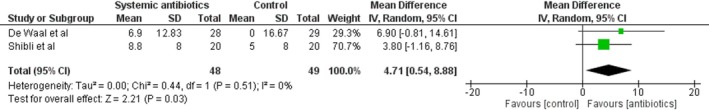
SOP reduction at 3 months in PI.

**FIGURE 10:**
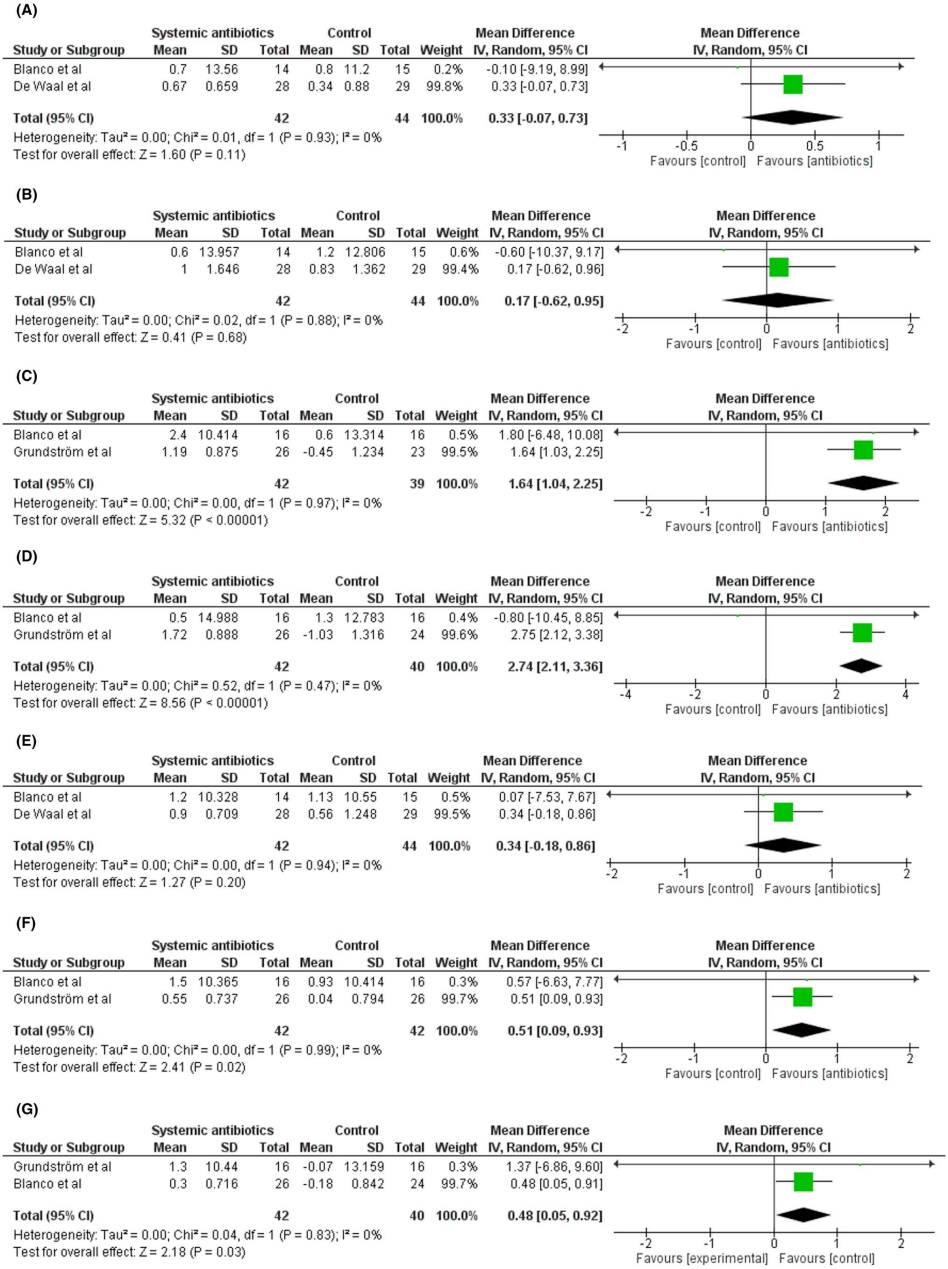
*F. nucleatum* reduction at 3 months (A) in PI; *P. gingivalis* reduction at 3‐ (B), 6‐ (C) and at 12‐months (D) in PI; *T. forsythia* reduction at 3‐ (E), 6‐ (F) and at 12‐months (G) in PI.

The meta‐analysis aimed to quantitatively synthesize the effects of SA therapy on various clinical and microbiological outcomes in patients with peri‐implant diseases (Figures 6, 7, 8, 9, 10).

#### 3.5.1 Clinical outcomes

The meta‐analysis of studies evaluating BOP revealed mixed findings depending on the different disease conditions and follow‐up duration. At 3 months, SA showed no statistically significant benefit over control treatments in reducing BOP for both PM and PI. Specifically, in PM (Figure 6A), the pooled MD was 1.34% (95% CI: −0.86% to 3.55%, *p* = 0.23), with no heterogeneity (I^2^ = 0%). In PI (Figure 6B), the estimated MD was 8.38% (95% CI: −6.80% to 23.55%, *p* = 0.28), with substantial heterogeneity (*I* ^2^ = 81%).

At 6 months (Figure 6C), no significant effect was observed either, with a pooled MD of 9.27% (95% CI: −1.79% to 29.32%, *p* = 0.37; *I* ^2^ = 87%). However, at 12 months (Figure 6D), a statistically significant reduction in BOP was detected in favor of SA, with an MD of 14.05% (95% CI: 3.77% to 24.33%, *p* = 0.007), and moderate heterogeneity (*I* ^2^ = 57%).

These results suggest that adjunctive SA therapy may provide a delayed benefit in BOP reduction, particularly evident at 12 months, while short‐term improvements at earlier timepoints remain inconsistent and inconclusive.

Adjunctive SA did not show a statistically significant benefit in terms of marginal bone gain at 3 months (Figure 7A; MD = 0.04 mm, 95% CI: −0.05 mm to 0.13 mm, *p* = 0.44, *I* ^2^ = 0%), 6 months (Figure 7B; MD = 0.42 mm, 95% CI: −0.13 mm to 0.98 mm, *p* = 0.14, *I* ^2^ = 0%) and 12 months (Figure 7C; MD = 0.60 mm, 95% CI: −0.30 mm to 1.51 mm, *p* = 0.19, *I* ^2^ = 82%). Overall, results did not confirm the potential benefits of SA in peri‐implantitis treatment, neither in the short term nor in the long term.

Regarding PM, at 3 months (Figure 8A), SA did not provide additional benefits on PPD reduction (MD = 0.24 mm, 95% CI: −0.11 mm to 0.60 mm, *p* = 0.18, *I* ^2^ = 0%). Instead, for PI, adjuvant SA produced a significant PPD reduction at 3 months (MD = 0.97 mm, 95% CI: 0.44 mm to 1.49 mm, *p* < 0.001, *I* ^2^ = 41%, Figure 8B), at 6 months (MD = 0.74 mm, 95% CI: 0.27 mm to 1.22 mm, *p* = 0.002, *I* ^2^ = 53%, Figure 8C) and at 12 months (MD = 0.84 mm, 95% CI: 0.26 mm to 1.41 mm, *p* = 0.004, *I* ^2^ = 57%, Figure 8D). This suggests a potential short‐term and delayed clinical effect of SA on peri‐implant PPD, even if the clinical gain benefit remained below 1 mm.

Only two studies provided extractable data on SOP reduction at 3 months (Figure 9). The pooled estimate showed a significant reduction in SOP favoring SA (MD = 4.71%, 95% CI: 0.54% to 8.88%, *p* = 0.03). These findings support the potential role of SA in controlling acute signs of infection in the short term, but the magnitude of the effect appears to be low.

#### 3.5.2 Microbiological outcomes

Data from two studies indicated no statistically significant reduction in *F. nucleatum* counts at 3 months with SA compared with controls (Figure 10A, MD = 0.33 log10 CFU, 95% CI: −0.07 to 0.73, *p* = 0.11, *I* ^2^ = 0%). Pooled data for *P. gingivalis* at 3 months (Figure 10B) showed no significant reduction with antibiotics. Conversely, at 6 months (Figure 10C, MD = 1.64 log10 CFU, 95% CI: −1.04 to 2.25, *p* < 0.001, *I* ^2^ = 0%) and at 12 months (Figure 10D, MD = 2.74 log10 CFU, 95% CI: 2.11 to 3.36, *p* < 0.001, *I* ^2^ = 0%) SA significantly reduced *P. gingivalis* peri‐implant load. For *T. forsythia*, no significant reductions were noted at 3 months (Figure 10E, MD = 0.34 log10 CFU, 95% CI: −0.18 to 0.86, *p* = 0.20, *I* ^2^ = 0%), whereas significant bacterial load reduction was observed at 6 months (Figure 10F; MD = 0.51 log_10_ CFU, 95% CI: 0.09 to 0.93, *p* = 0.02, *I* ^2^ = 0%) and at 12 months (Figure 10G; MD = 0.48 log10 CFU, 95% CI: 0.05 to 0.92, *p* = 0.03, *I* ^2^ = 0%). These findings suggest that, while some benefits exist for the long‐term suppression of specific pathogens such as *T. forsythia* and P. *gingivalis*, the overall microbiological improvements around peri‐implant tissues appear to be inconsistent, especially in the short term.

## 4 DISCUSSION

This systematic review and meta‐analysis critically evaluated the role of adjunctive SA in the treatment of peri‐implant diseases, with particular attention to their clinical and microbiological effects across short‐ and long‐term follow‐up periods of PM and PI. According to the findings, for the PM management, it appears that SA did not significantly affect BOP and PPD. On the other hand, in patients with PI, adjunctive SA produced mild additional benefits on BOP, SOP, and PPD up to 12 months, whereas they were not effective in improving MBL compared with controls. Moreover, SA regimens significantly reduced *P. gingivalis* and *T. forsythia* levels at 6 and 12 months follow‐up in PI.

From a clinical point of view, it has been shown that, after nonsurgical or surgical PI treatment, residual deep peri‐implant pockets with (PPD > 5 mm) and BOP are predictors of disease progression.^88^, ^89^, ^90^ Moreover, a previous meta‐analysis showed that around one third of BOP‐positive implants have been affected by PI.^91^, ^92^, ^93^, ^94^ According to the findings of the present meta‐analysis, adjunctive SA were ineffective in reducing BOP at 3 and 6 months. However, a modest significant peri‐implant BOP reduction (% sites) at 12 months (MD = 14.05%), SOP reduction at 3 months (MD = 4.71%), and PPD reduction at 3 months (MD = 0.97 mm), 6 months (MD = 0.74 mm), and 12 months (MD = 0.84 mm) was observed compared with controls. These results suggest that SA may be beneficial in determining a significant improvement in peri‐implant inflammation. However, these findings should be interpreted cautiously given the moderate heterogeneity and variation in treatment protocols. Moreover, no significant gains in marginal bone levels were observed at any time point, questioning the effectiveness of SA on peri‐implant bone loss.

Regarding microbiological outcomes, the meta‐analysis revealed statistically significant reductions in levels of *P. gingivalis* and *T. forsythia* at 6 and 12 months in patients with PI but no significant differences in the short‐term (3 months) were observed compared with controls. Nevertheless, several studies lacked extractable numerical data for meta‐analysis.^74^, ^80^, ^81^, ^84^, ^87^ Specifically, results from the other studies^16^, ^17^, ^76^ reported that in the 1 month and 3 months after the administration of SA, there was a reduction in peri‐implant bacteria levels, including *F. nucleatum*, *P. gingivalis*, *P. intermedia*, *T. denticola*, and *T. forsythia*, whereas their expression often increased again over follow‐up sessions, supporting the hypothesis that SA could be able to influence subgingival biofilm composition, especially in the short term. However, the contrasting results across studies limit the generalisability of microbiological outcomes, highlighting the transient nature of these improvements in the absence of sustained mechanical or surgical debridement.

However, the clinical relevance of these effects must be weighed against their potential drawbacks.^61^, ^95^ For instance, the study by Gomi et al.^80^ consistently appeared as an outlier in the meta‐analyses. This may be explained by its small sample size and high risk of bias, together with relevant protocol differences, including preoperative azithromycin administration and the inclusion of patients affected by both chronic periodontitis and peri‐implantitis. Moreover, outcomes were reported at the implant level without adjustment for clustering. These factors may have contributed to larger effect estimates and increased heterogeneity. Overall, despite possible modest improvements in clinical and microbiological short‐term peri‐implant outcomes, SA does not appear to provide clinically significant benefits in inflammation control or tissue regeneration in the long term. Furthermore, concerns regarding bacterial resistance, side effects, and ecological disruption of the oral microbiome suggest that SA should not be routinely prescribed for peri‐implant diseases. For instance, according to a previous review of studies,^96^ it was reported that *P. gingivalis* and *F. nucleatum* mainly could present high resistance to certain SA such as tetracycline, MTZ, and erythromycin in patients with PI.

Similarly, *A. actinomycetemcomitans* was also highly resistant to clindamycin and doxycycline, whereas *T. forsythia*, *P. micra*, and *P. intermedia/nigrescens* also presented significant levels of resistance to AMX, AZT, and moxifloxacin. According to the findings of another study,^21^ 71.7% of the 120 PI subjects exhibited submucosal bacterial pathogens resistant in vitro to one or more of the tested SA, among which doxycycline, AMX, MTZ, and clindamycin. Therefore, given the potential for bacterial resistance and its broad impact on the human microbiome, careful consideration of the empirical administration of SA is essential to maximize patient benefit and reduce related potential side effects. Furthermore, in line with the long‐term outcomes observed in the present analysis, evidence from well‐designed RCTs indicates that the adjunctive use of systemic antibiotics does not confer sustained clinical or radiographic benefits beyond the first year following surgical PI therapy. In particular, the 3‐year follow‐up study by Carcuac et al.^77^ demonstrated that adjunctive systemic amoxicillin did not result in additional long‐term improvements in PPD reduction, BOP, or SOP, or marginal bone level stability when compared with surgery alone. Although transient benefits were reported during the initial healing phase—mainly at implants with modified surface characteristics—these effects were not maintained at 3 years. Collectively, these findings support the interpretation that SA should not be expected to enhance long‐term disease resolution but may still play a role in maximizing early surgical outcomes by improving initial infection control and limiting the risk of postoperative complications.^4^

In this context, parallels can be drawn with SA during periodontal therapy. According to the 2019 European Federation of Periodontology (EFP) S3‐level clinical practice guidelines on stage I–III periodontitis, SA are reserved for severe and rapidly progressing periodontitis (e.g., generalized periodontitis Stage III in young adults), since a prolonged impact on the fecal microbiome, specifically an increase in antimicrobial resistance‐associated genes, has been observed following SA regimens.^97^ A similar conservative approach should guide PI treatment. In this regard, the EFP S3‐ level clinical practice guidelines on peri‐implant diseases^4^ do not recommend prescribing SA for PM due to concerns about patients' and public health. Among the other, the findings of this meta‐analysis confirmed no significant additional benefits of adjuvant SA in PM‐related clinical and microbiological outcomes. On the other hand, in PI patients, the EFP expert group did also not recommend the routine use of SA as an adjunct to nonsurgical treatment, but it should be limited to cases at the end of the severity spectrum (e.g., deep pockets ≥7 mm, extensive suppuration) and/or with multiple and/or strategically affected implants that could respond well and be retained over time.^4^ In accordance, the marginal benefits observed in the present study are insufficient to recommend SA for general use in PI, especially given the absence of clear indications and standardized regimens across studies.

This study also identified important limitations in the analyzed literature on the topic. Included studies differed substantially in design, population, antibiotic regimens (type, dose, duration), and comparator groups. Some studies employed placebo controls or only conventional treatment without placebo; others lacked any comparator. The nature of the baseline treatment (mechanical versus surgical debridement) also varied, introducing further heterogeneity. Clinical outcomes were inconsistently reported at either the patient or the implant level, and microbiological data were often graphically displayed without numerical extractability. Risk of bias was high or unclear in several trials, particularly regarding randomization, blinding, and selective outcome reporting.

Based on the abovementioned evidence, the present meta‐analysis shows that for PM, adjunctive SA do not provide additional clinical or microbiological benefits compared with mechanical debridement alone. Reductions in inflammatory parameters observed in both test and control groups appear to be primarily attributable to biofilm removal and improvements in oral hygiene rather than to antimicrobial administration. Therefore, the routine use of systemic antibiotics in the management of PM is not supported by current evidence.

In contrast, for PI, adjunctive systemic antibiotics may confer modest short‐ to medium‐term benefits for selected clinical parameters. The meta‐analysis showed statistically significant reductions in BoP at 12 months, suppuration on probing at short‐term follow‐up, and PPD at 3, 6, and 12 months when SA were used in conjunction with nonsurgical or surgical therapy. However, these improvements were not consistently associated with radiographic benefits, as no significant adjunctive effect on MBL changes was demonstrated across follow‐up periods. From a microbiological perspective, adjunctive systemic antibiotics were associated with reductions in selected periodontal pathogens, notably *P. gingivalis* and *T. forsythia*, in some studies on PI. Nevertheless, these effects were inconsistent across trials, often transient, and not uniformly correlated with superior clinical outcomes.

The limited number of studies providing quantitative microbiological data and the heterogeneity of analytical methods further restrict the strength of these conclusions. In this regard, a possible limitation of the present study relates to the methodological constraints inherent to quantitative data synthesis. The results of the performed meta‐analyses should be interpreted with caution, as pooling data from different studies is methodologically robust only when a minimum number of comparable studies is available. In line with contemporary evidence‐based standards, meta‐analyses based on fewer than three studies are increasingly considered questionable due to limited statistical power and an increased risk of biased estimates. This methodological principle has been consistently adopted in recent EFP guidelines^4^ and reflects a growing emphasis on rigor and reproducibility in periodontal and peri‐implant research. Consequently, although quantitative synthesis was undertaken when feasible, the conclusions drawn from meta‐analyses including a limited number of studies should be regarded as exploratory rather than definitive.

## 5 CONCLUSIONS

Based on the results of the present meta‐analysis, SA should not be recommended for PM and may be considered only as an adjunctive measure in selected PI cases, preferably within a comprehensive therapeutic protocol and after careful patient‐ and site‐specific risk assessment. Alternative strategies, such as local antimicrobials, host modulation therapies, or precision‐based regimens, may be more effective and safer adjuncts to mechanical treatment.

However, given the heterogeneity of the available evidence, the generally high risk of bias, and concerns about adverse events and antimicrobial resistance, SA should be reserved for carefully selected PI cases within a comprehensive, personalized treatment strategy. Further well‐designed randomized controlled trials with standardized outcomes and long‐term follow‐up are required to clarify their true clinical value, especially in the long‐term.

## AUTHOR CONTRIBUTIONS

Gaetano Isola conceived the research, planned and performed the literature review, and wrote the manuscript. Alessandro Polizzi performed the article screening. Angela Angjelova and Elena Jovanova performed the literature search. Giuseppe Pizzo critically reviewed the manuscript. Anton Sculean validated the results and wrote the manuscript. All the authors gave their final approval and agreed to be accountable for all aspects of the work.

## FUNDING INFORMATION

The study was supported by a grant from the Italian Ministry of Health, Principal Investigator Prof. G. Isola, grant no. PNRR‐POC‐2023‐12 377 354.

## CONFLICT OF INTEREST STATEMENT

The authors declare no conflict of interest in the present report.

## Data Availability

Data are available from the corresponding author upon reasonable request.
